# *Chlamydia trachomatis* development requires both host glycolysis and oxidative phosphorylation but has only minor effects on these pathways

**DOI:** 10.1016/j.jbc.2022.102338

**Published:** 2022-08-02

**Authors:** Maimouna D. N’Gadjaga, Stéphanie Perrinet, Michael G. Connor, Giulia Bertolin, Gaël A. Millot, Agathe Subtil

**Affiliations:** 1 Institut Pasteur, CNRS UMR3691, Cellular Biology of Microbial Infection, Université Paris Cité, Paris, France; 2 Sorbonne Université, Collège Doctoral, Paris, France; 3 Institut Pasteur, Chromatin and Infection, Université Paris Cité, Paris, France; 4 CNRS, IGDR (Institute of Genetics and Development of Rennes), UMR 6290, Univ Rennes, Rennes, France; 5 Institut Pasteur, Hub Bioinformatique et Biostatistique–DBC, Université Paris Cité, Paris, France

**Keywords:** metabolism, glycolysis, oxidative phosphorylation, ATP, host-pathogen interaction, *Chlamydia trachomatis*, ACC, acetyl-CoA carboxylase, CTD, *C. trachomatis* serovar D, EB, elementary body, ECAR, extracellular acidification rate, FLIM, Fluorescence Lifetime Imaging Microscopy, G6P, glucose-6-phosphate, gDNA, genomic DNA, GPI, glucose phosphate isomerase, hpi, hours postinfection, IFU, infection forming unit, LDH, lactate dehydrogenase, MTOC, microtubule organizing center, OCR, oxygen consumption rate, OxPhos, oxidative phosphorylation, qPCR, quantitative PCR, RB, reticulate body

## Abstract

The obligate intracellular bacteria *Chlamydia trachomatis* obtain all nutrients from the cytoplasm of their epithelial host cells and stimulate glucose uptake by these cells. They even hijack host ATP, exerting a strong metabolic pressure on their host at the peak of the proliferative stage of their developmental cycle. However, it is largely unknown whether infection modulates the metabolism of the host cell. Also, the reliance of the bacteria on host metabolism might change during their progression through their biphasic developmental cycle. Herein, using primary epithelial cells and 2 cell lines of nontumoral origin, we showed that between the 2 main ATP-producing pathways of the host, oxidative phosphorylation (OxPhos) remained stable and glycolysis was slightly increased. Inhibition of either pathway strongly reduced bacterial proliferation, implicating that optimal bacterial growth required both pathways to function at full capacity. While we found *C. trachomatis* displayed some degree of energetic autonomy in the synthesis of proteins expressed at the onset of infection, functional host glycolysis was necessary for the establishment of early inclusions, whereas OxPhos contributed less. These observations correlated with the relative contributions of the pathways in maintaining ATP levels in epithelial cells, with glycolysis contributing the most. Altogether, this work highlights the dependence of *C. trachomatis* on both host glycolysis and OxPhos for efficient bacterial replication. However, ATP consumption appears at equilibrium with the normal production capacity of the host and the bacteria, so that no major shift between these pathways is required to meet bacterial needs.

Lifestyles of intracellular bacteria exert strong metabolic pressure upon their host through acquisition of cytoplasmic nutrients during replication. The obligate intracellular bacteria *Chlamydia trachomatis* provides an extreme illustration of this situation by relying on the host not only for the supply of glucose, their main carbon source (1), but also for ATP.

One singularity of the chlamydiae phylum is the reversible differentiation between 2 morphologies with unique metabolic capacities and requirements during the intracellular lifecycle. The infectious form, called the elementary body (EB), shows little metabolic activity, consistent with the absence of replication. Experiments using axenic medium demonstrated that protein synthesis in EBs required the supply of glucose-6 phosphate (G6P), not ATP (2). EBs contain glycogen (3), which is thought to be the source of energy in the first hours of infection, prior to differentiation into the replicative form, called the reticulate body (RB). In contrast, G6P does not support protein synthesis in RBs, while ATP does (2). This is not due to a lack of G6P import, since a proteomic study showed that the G6P transporter, UhpC, is actually more abundant in RBs than in EBs (4). In fact, the same study revealed that the expression of glycolytic enzymes is very low in RBs compared to EBs, suggesting that in RBs, glucose is not primarily used to feed ATP production through glycolysis. Indeed, Mehlitz *et al.* elegantly demonstrated that G6P was mostly shuffled into the production of lipooligosaccharide (5). For energy supply, RBs are thought to rely on their capacity to take up ATP from the host through the expression of the ADP/ATP translocase, Ntp1, and the ribonucleotide triphosphate importer Ntp2 that are very abundant in RBs, not in EBs (4, 6).

Regarding other metabolic pathways, the bacteria also highly depend on the host to synthesize many essential metabolites. They exert, in particular, a strong demand on amino acids and nucleotides. For the latter, they rely exclusively on their host, having lost the genes of nucleotide biosynthesis (7). For the former, they show little *de novo* amino acid production and import most of them from the host (5). Highlighting this, glutamine was recently shown to be essential for peptidoglycan synthesis (8). Finally, the bacteria appear less dependent on the host for lipid biosynthesis, as the organism has retained all of the genes required for fatty acid and phospholipid synthesis (9), however, some scavenging of host lipids has been shown to occur (10, 11).

Overall, the metabolic demand exerted by *C. trachomatis* on the host cell evolves across the infectious cycle. It is not known whether the glycogen reserve provides sufficient autonomy to establish the infectious niche and start replication. Importantly, in the first hours of infection, bacterial biomass is small compared to that of their host, therefore, their metabolism does not represent a significant burden for the host cell (Fig. S1*A*). However, by 20 h postinfection (hpi), there is an average of >300 bacteria per cell (12). With about 10^6^ base pairs (bp) for the bacterial genome against 3 × 10^9^ bp for the host, at that stage of infection, bacterial DNA represents about 10% of total DNA in an infected cell, and demand in ATP, amino acids, glucose becomes significant. Twenty hpi is about the time when RBs begin conversion into EBs, with a parallel and gradual shift toward lower nutritional need per bacteria. Importantly, because the conversion of RBs into RBs is asynchronous, the number of RBs continues to increase and so does the metabolic burden. It is alleviated only after ∼30 to 32 hpi, when the number of RBs per inclusion starts decreasing (13). Glucose demands also probably decrease by that time, because the bacteria obtain it from the degradation of the glycogen that has accumulated in the inclusion lumen during the first half of the developmental cycle (3).

How does the host cell cope with this increasing and then decreasing metabolic burden? Since hijacking ATP is central to *C. trachomatis* metabolic strategy, we investigated here the 2 main sources of ATP in the host cell, glycolysis and oxidative phosphorylation (OxPhos). Specifically, we addressed whether infected cells modified their glycolysis or OxPhos capacities and at what stage of the infectious cycle were these host pathways critical for optimal bacterial replication. Past work had shown that glycolysis was important for bacterial development (14, 15). However, these studies were conducted mainly in cancer-derived cell lines, which are known to have an abnormal metabolism. Ideally, metabolic studies should use primary cells. However, primary epithelial cells divide only a few times, yielding limited numbers of cells, and are thus not amenable to some metabolomic studies. Furthermore, they are not very permissive to transfection techniques, precluding overexpression or gene silencing strategies. To circumvent these difficulties, we privileged in the present study the use of primary cells isolated from the human ectocervix and of A2EN cells, an immortalized epithelial cell line derived from the human endocervix (16). Regarding the bacterial strain, we chose to use *C. trachomatis* serovar D (CTD), as it is a reference strain more representative of circulating strains than the more commonly used strain LGV serovar L2.

## Results

### Cell attachment and growth are enhanced in stably transformed *C. trachomatis* serovar D bacteria

**Figure 1:**
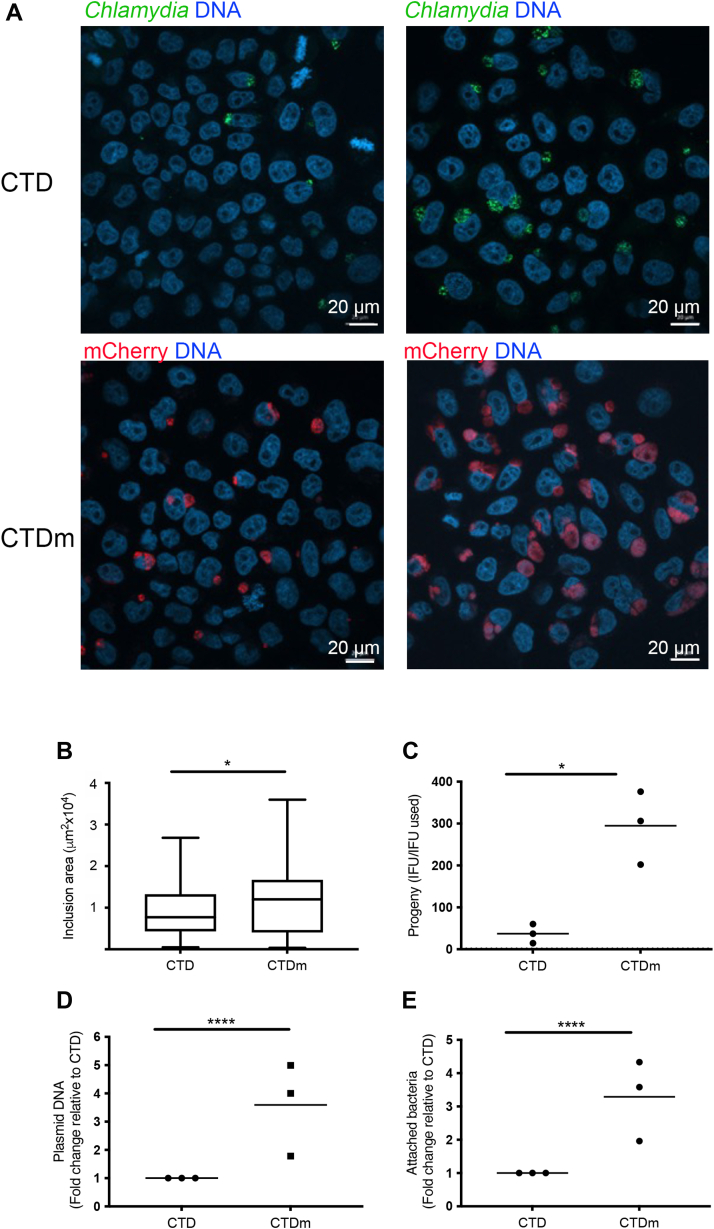
**CTDm is more infectious than CTD.***A*, HeLa cells were infected with CTD or CTDm, centrifuged (right) or not (left) and fixed 24 hpi. CTD-infected cells were permeabilized and immunostained with antibodies against *Chlamydia* followed with Alexa488-conjugated secondary antibodies (*green*). DNA is stained with Hoechst. *B*, CTD and CTDm inclusion size was measured with the CellProfiler software. The graph displays the mean ± SD (n = 38 inclusions/condition). *C*, HeLa cells were infected with equal IFUs of CTD and CTDm for 48 h. Cells were lysed and bacterial IFU were determined by reinfecting fresh HeLa cells as described in the Experimental procedures. The result of 3 independent experiments and the mean are displayed. *D*, total DNA was extracted from CTD and CTDm EBs. Bacterial plasmidic DNA (pDNA) was measured by qPCR and was normalized to bacterial genomic DNA (gDNA). The data are presented as the fold change pDNA over gDNA in CTDm relative to CTD. The results of 3 independent experiments, each performed in triplicates, and the mean are displayed. *E*, precooled HeLa cells were infected with equal genome number CTD and CTDm at 4 °C, then incubated for 3h30 at 4 °C. HeLa cells were then gently washed with cold PBS then lysed for total DNA extraction. Bacterial gDNA was measured by qPCR and was normalized to human gDNA. The results of 3 independent experiments, each performed in triplicates, and the mean are displayed. Statistical analyses were done using Welch tests. See the Experimental procedures section and Table S1 for details of the statistical analysis of each figure. Significance is defined as: (∗) = *p* ≤ 0.05; (∗∗∗∗) = *p* ≤ 0.0001. CTD, *C. trachomatis* serovar D; EB, elementary body; hpi, hours postinfection; IFU, infection forming unit; qPCR, quantitative PCR.

The *C. trachomatis* D/UW-3/CX reference strain was stably transformed with a L2-based plasmid encoding the fluorescent protein mCherry under the control of the promoter of the early gene *incD* (17). Expressing mCherry allowed quantitative tracking by flow cytometry: mCherry expressing bacteria (CTDm) detached from the noninfected population around 18 hpi, with a gradual increase in the fluorescent signal with time (Fig. S1*B*). We observed that even without a centrifugation step classically applied to facilitate infection with serovars of *C. trachomatis* others than LGV, we obtained good infection rates when using CTDm. When normalizing the stocks to an equal genome number, we observed both an increase in the number and size of inclusions in cells infected with CTDm compared to CTD (Fig. 1, *A* and *B*). This suggested that bacteria gained in infectivity upon stable transformation. To test this, we quantified the infection forming units (IFUs) obtained in cells infected with equal genome equivalent of the parental and of the stably transformed strain. CTDm gave rise to ∼8 fold more IFU than the parental CTD strain (Fig. 1*C*).

The plasmid used for the transformation is the natural plasmid of *C. trachomatis*, shown to provide a selection advantage over plasmid-free bacteria *in vitro* and *in vivo* (18). We hypothesized that selection of plasmid-transformed bacteria may have increased the quantity of plasmid copies per bacteria. Indeed, using unique quantitative PCR (qPCR) primers to the plasmid or to the bacterial chromosome, we showed that CTDm bacteria displayed ∼3 fold more plasmid copies per bacteria than the parental strain (Fig. 1*D*). This paralleled a ∼3-fold increase in the adhesion capacity of the fluorescent strain (Fig. 1*E*), consistent with the increase in the percentage of infected cells when using CTDm compared to CTD (Fig. 1*A*). Thus, stable transformation increases plasmid number and favors adhesion. Moreover, by 24 hpi, CTDm inclusions were about 30% larger than CTD inclusions, indicating that the selection of stable transformants also provides an advantage to intracellular growth rate (Fig. 1*B*). Altogether, CTDm bacteria represent a convenient model to follow *C. trachomatis* serovar D development, due to increased traceability of bacterial growth *in vitro*. Regarding the metabolic requirements during the course of infection, it is expected that the pressure exerted by the bacteria on the host metabolism occurs slightly earlier in time than with the parental strain since the stably transformed bacteria multiply faster.

### ATP levels remain largely stable in epithelial cells infected by *C. trachomatis*

**Figure 2:**
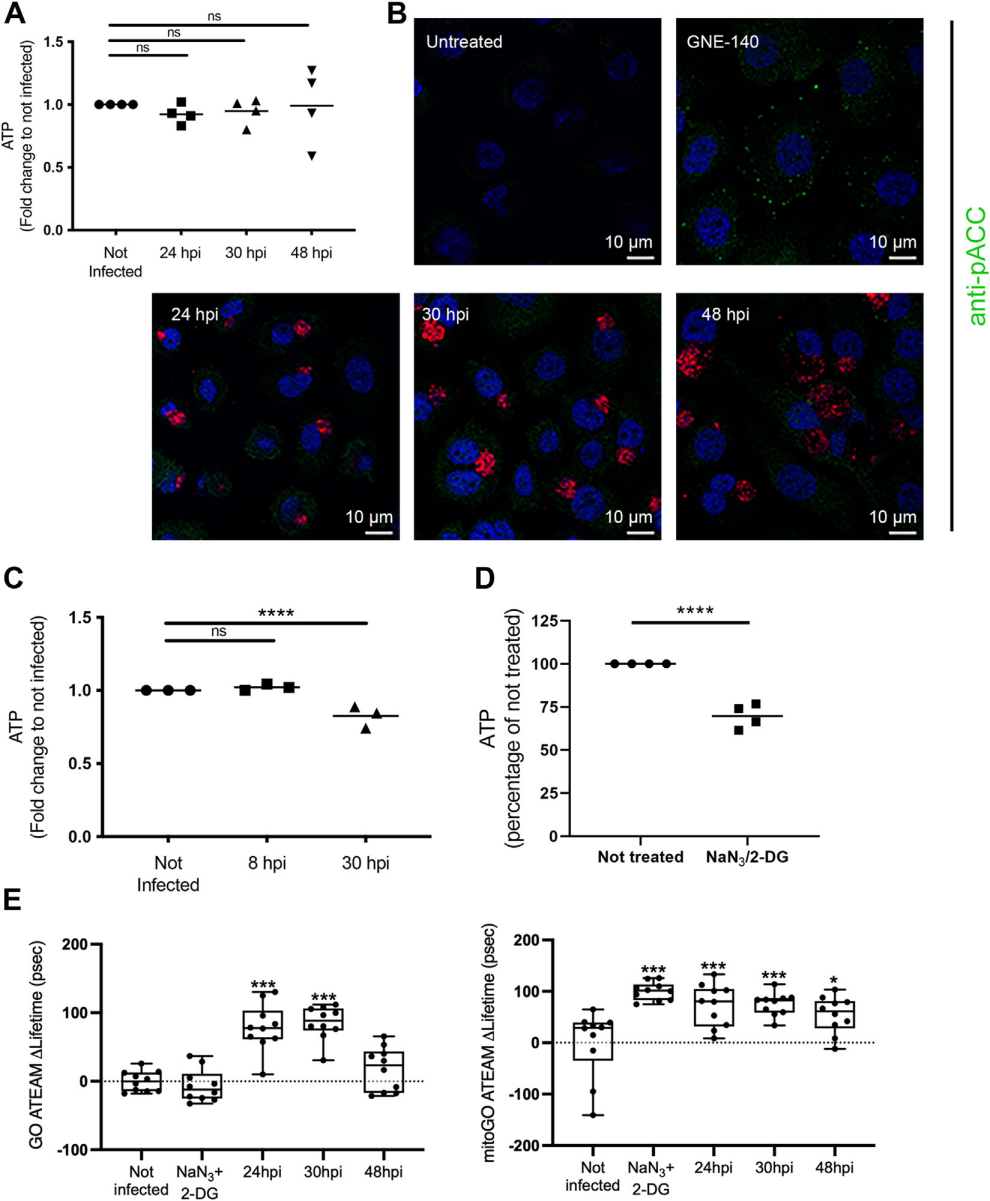
**ATP levels remain stable over the course of *Chlamydia trachomatis* infection in epithelial cells.***A*, primary cells were infected with CTDm and intracellular ATP was measured 24, 30, and 48 hpi. *B*, A2EN cells were treated for 2 h with 10 μM GNE-140 or infected for the indicated time with CTDm before fixation, permeabilization, and staining with antibodies against the phosphorylated form of ACC (*green*). Bacteria (mCherry) are *red*, and nuclei (DAPI) are *blue*. *C*, ATP levels were measured in CTDm-infected HeLa cells at 8 and 30 hpi. *D*, ATP levels were measured in HeLa cells 1 h after treatment with 10 mM 2-deoxyglucose (2-DG) and 0.019% NaN_3_. *E*, HeLa cells were transfected with plasmids expressing GO-ATEAM2 (*left*) and mitoGO-ATEAM2 (*right*) for 24 h, then infected with CTDm for the indicated time before fixation and image analysis as described in the Experimental procedures section. An increase in the ΔLifetime corresponds to a drop in FRET and thus a decrease in ATP concentration. One hour incubation in 10 mM 2-DG and 0.019% NaN_3_ was used as a positive control. Significance is defined as: ns (not significant) *p* > 0.05; (∗) = *p* ≤ 0.05; (∗∗∗) = *p* ≤ 0.001; (∗∗∗∗) = *p* ≤ 0.0001. ACC, acetyl-CoA carboxylase; CTD, *C. trachomatis* serovar D; hpi, hours postinfection.

**Figure 5:**
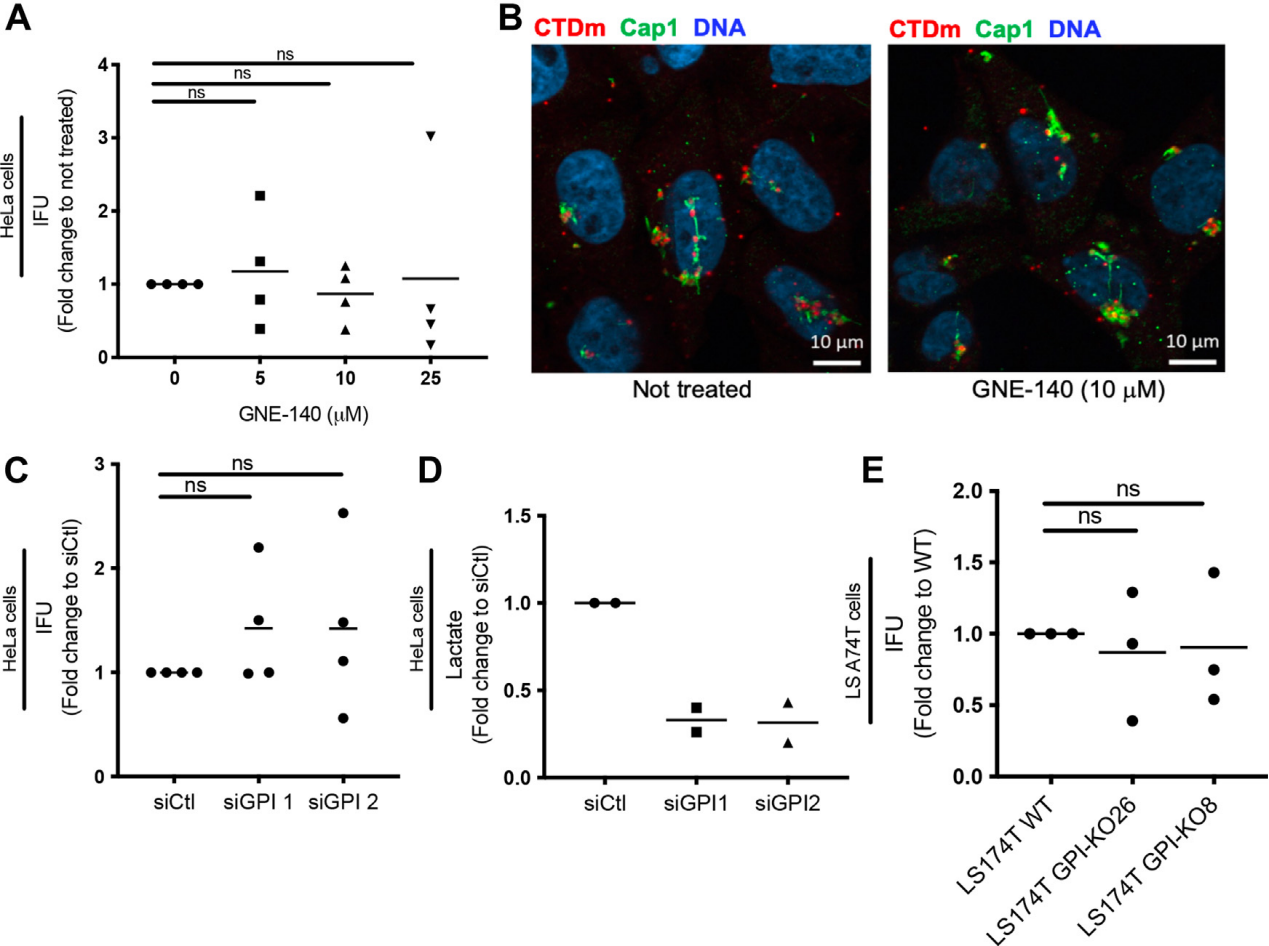
**Reliance of *Chlamydia trachomatis* on host glycolysis is by-passed in 2 tumor-derived cell lines.***A*, HeLa cells were infected with CTDm and treated with indicated concentrations of GNE-140 for 48 h before a progeny assay was performed as described in the Experimental procedures, and IFU was determined. The results of 4 independent experiments and the mean are displayed. *B*, HeLa cells were infected at MOI 10 with CTDm and treated or not with GNE-140. Cells were fixed 6 hpi, then permeabilized before immunostaining with anti-Cap1 followed with Alexa488-conjugated secondary antibodies. DNA is stained with Hoechst. *C*, GPI was silenced in HeLa cells with 2 siRNA for 48 h before cells were infected with CTDm. 30 hpi, a progeny assay was performed, and IFU was determined. The results of 4 independent experiments and the mean are displayed. *D*, efficiency of siRNA treatment was evaluated through a lactate assay 48 h following siRNA treatment in HeLa cells. The results of 2 independent experiments and the mean are displayed. *E*, LS174T WT or GPI-KO (clone #26 and #8) were infected with CTDm for 48 h before performing a progeny assay and determining IFU. The results of 3 independent experiments and the mean are displayed. CTD, *C. trachomatis* serovar D; IFU, infection forming unit; hpi, hours postinfection.

To gain an overall picture of the energetic balance of infected cells, we first measured the concentration of ATP over 48 h in primary epithelial cells using a luciferase-based assay emitting a luminescent signal proportional to the ATP concentration. This assay measures total ATP, including the ATP pool in the bacteria. Strikingly, ATP levels remained stable over the course of infection (Fig. 2*A*). To monitor a potential drop in ATP level in the host cytoplasm during infection, we stained cells for phosphorylated acetyl-CoA carboxylase (ACC). ACC is a substrate of AMP-activating protein kinase that phosphorylates and inactivates ACC when ATP levels are diminished (19). As a positive control, we treated cells with 10 μM of the lactate dehydrogenase (LDH) A/B inhibitor GNE-140, which decreases ATP level by inhibiting glycolysis (see Fig. 5*B*). As expected, GNE-140 treatment resulted in an increase in the phosphorylated form of ACC detected by fluorescence microscopy (Fig. 2*B*). In contrast, the phosphorylated form of ACC level in infected cells remained low and undistinguishable from the surrounding noninfected cells at all time points examined (Fig. 2*B*). Overall, these experiments indicated that the ATP level in primary epithelial cells remained stable during *C. trachomatis* infection.

Previously, Kurihara *et al.* (2019) demonstrated that *C. trachomatis* serovar L2 infection induced an increase of ATP levels in the cancer-derived HeLa cell line, by 8 hpi (20). Using the CTDm strain, we did not observe any increase in ATP level 8 hpi in HeLa cells, but we observed a drop at 30 hpi (Fig. 2*C*). To determine whether the drop in total ATP levels in CTDm-infected HeLa cells was attributed to the host or the bacteria, we used FRET-based probes specific to ATP. GO-ATEAM2 is a probe that measures variations in cytosol ATP levels, while mitoGO-ATEAM2 is targeted to mitochondria and is sensitive to mitochondrial ATP concentration (21). We calculated FRET using Fluorescence Lifetime Imaging Microscopy (FLIM), and we used change in probe lifetime (ΔLifetime) to indicate net lifetime differences between the controls and all other conditions. A positive net ΔLifetime corresponds to a drop in FRET and thus a decrease in ATP concentration in their respective compartment. HeLa cells were transfected with plasmids expressing these probes and infected at different times, to synchronize cell fixation at 24, 30, or 48 hpi. As a positive control, we treated cells for 1 h with 10 mM 2-deoxyglucose and 0.019% sodium azide, which decreased total ATP concentration by 30% (Fig. 2*D*). We observed a significant increase in ΔLifetime for the mitochondrial probe upon treatment with sodium azide and 2-deoxyglucose, while having no significant effect on the cytosolic probe. During infection with CTDm, an increase in the ΔLifetime of both probes was observed. However, at 48 hpi, it was significant only with the mitochondrial probe (Fig. 2*E* and S2). We concluded from these experiments that HeLa cells undergo a decrease in ATP concentration during infection, affecting both mitochondrial and cytosolic pools.

### Glycolysis increases slightly, while OxPhos remains stable in epithelial cells infected by *C. trachomatis*

Total ATP concentration results from the balance between production and consumption, by both the host and the bacteria. The stability in total ATP levels in primary cells suggested that ATP supply and demand requirements during CTDm development, even at the peak of bacterial proliferation, was modulated to maintain a total pool of host ATP. Thus, we investigated the rates of glycolysis and OxPhos in the host separately during infection to determine if a compensatory effect of these pathways was exploited to maintain host ATP levels.

**Figure 3:**
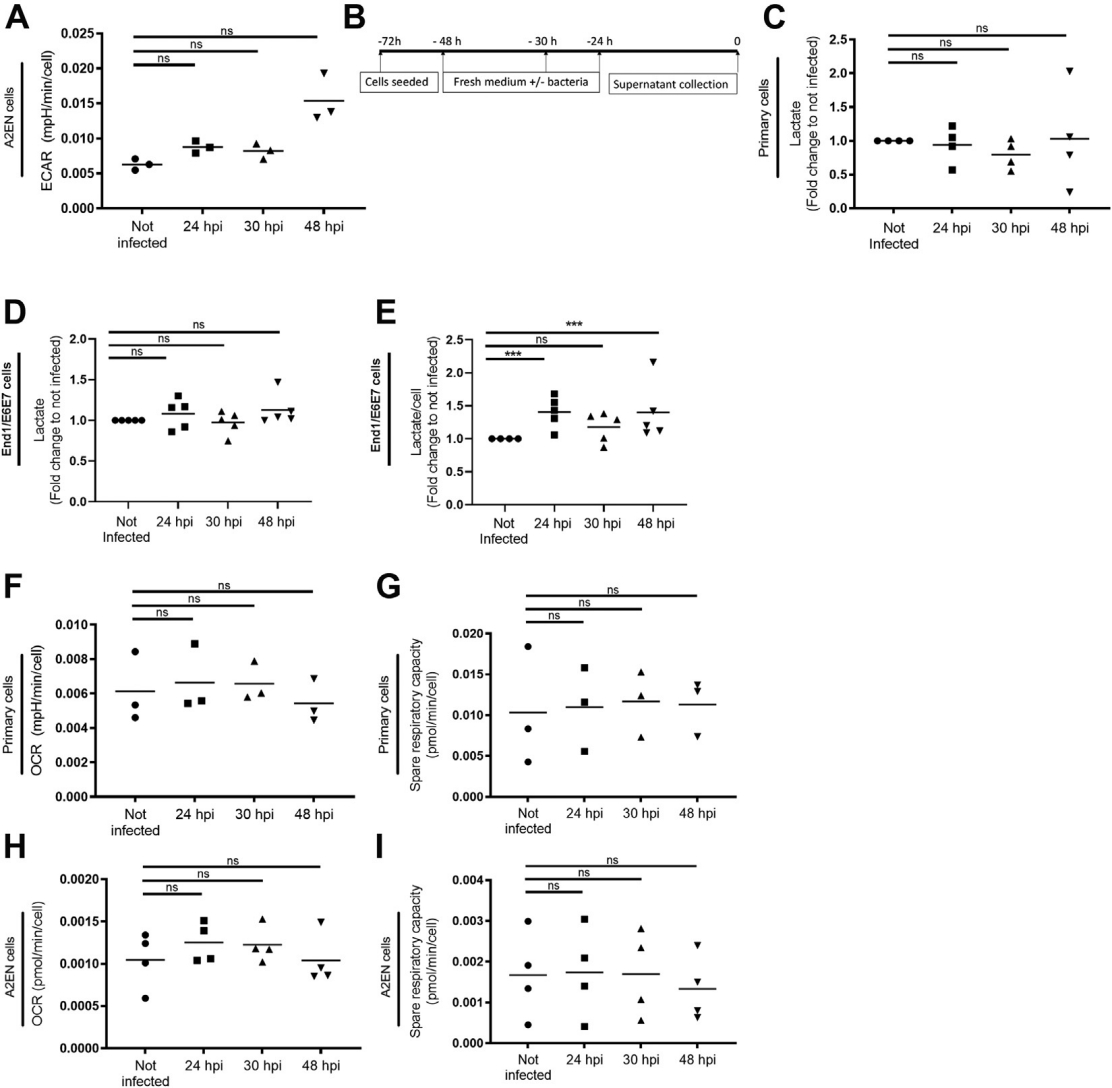
**Glycolysis and OxPhos remain largely stable over the course of *Chlamydia trachomatis* infection in epithelial cells.***A*, A2EN cells were infected with CTDm for 24, 30, and 48 h followed by ECAR measurements as described in the Experimental procedures. Note that the increase in ECAR observed 48 hpi was no longer significant after Benjamini Hochberg adjustment of the *p* values, see Table S1 for details. *B*, experimental setup for the measurement of lactate concentration in the culture medium. *C*, primary cells (*C*) and End1 cells (*D* and *E*) were infected with CTDm for 24, 30, and 48 h after what the concentration of lactate in the culture medium was measured, without (*C* and *D*) or with (*E*) normalization to the cell number. Primary cells (*F* and *G*) and A2EN cells (*H* and *I*) were infected with CTDm for 24, 30, and 48 h followed by OCR and spare respiratory capacity measurements as described in the Experimental procedures (see also Fig. S3*B*). For each panel, the results of 3 or 4 independent experiments and the mean are displayed. Significance is defined as: ns (not significant) *p* > 0.05; (∗∗∗) = *p* ≤ 0.001. CTD, *C. trachomatis* serovar D; ECAR, extracellular acidification rate; hpi, hours postinfection; OCR, oxygen consumption rate; OxPhos, oxidative phosphorylation.

Glycolysis is commonly determined through measurements of the extracellular acidification rate (ECAR) of the surrounding media (Fig. S3*A*). The latter is mostly governed by the rate of excretion of lactic acid, itself a by-product of the glycolytic pathway. Importantly, *C. trachomatis* lacks LDH and does not contribute to lactic acid production. Thus, even in the context of the infection, it might be possible to use ECAR as a readout of the glycolytic activity of the host cell. Herein, we compared the ECAR of the immortalized epithelial endocervical cell line A2EN infected or not with CTDm for 24, 30, or 48 h. ECAR remained comparably stable for the first 30 h of infection, when the energy demand of the bacteria is estimated to be at its peak and showed a trend toward an increase late in infection (Fig. 3*A*).

ECAR measurements require a substantial number of cells. To validate these findings in primary epithelial cells, we chose to directly measure lactate quantities in the supernatant of cell cultures, infected or not with CTDm, a technique that is less demanding on cell quantities. The second advantage of this readout is that lactate can only be produced by the host and thus it constitutes a more direct readout of glycolysis in the host than ECAR. Cells were seeded in 48-well plates and infected or not at different times to collect all supernatants simultaneously (Fig. 3*B*). No difference was seen between lactate concentrations in the culture medium of infected and noninfected primary cells (Fig. 3*C*). However, this assay was not sensitive enough to measure lactate production by A2EN. To confirm the stability in lactate production in another cellular background than primary cells, we used the End1/E6E7 cell line, an epithelial cell line also derived from the endocervix. In these series of experiments, we fixed cells at the time of collection of the culture supernatants, to quantify cell numbers per well by microscopy. As in primary cells, the amount of lactate produced in each well was not significantly different between infected and noninfected wells (Fig. 3*D*). When lactate production was expressed relative to cell number, quantified by microscopy, a 40% increase was observed at 24 and 48 hpi but not at 30 hpi (Fig. 3*E*). An increase in lactate production was also observed in HeLa cells at 48 hpi but not at earlier times (Fig. 3, *C* and *D*).

In summary, our data indicate that the glycolytic pathway tends to increase in epithelial cells infected by *C. trachomatis* serovar D and this is mostly significant at the end of the developmental cycle.

The level of OxPhos in eukaryotic cells is commonly measured by following oxygen consumption rate (OCR, Fig. S3*B*). *C. trachomatis* has an active respiratory metabolism, and it has been estimated that up to 40% of the OCR in infected cells might represent bacterial oxygen consumption (22). Thus, basal OCR cannot be used as a readout of the level of OxPhos in the host. However, the respiratory chain in *C. trachomatis* is very unusual, as it uses a sodium-linked respiratory chain to produce ATP (22). Addition of oligomycin, an inhibitor of the ATP synthase (complex V) of the respiratory chain of the host, allows to measure the mitochondrial respiration, as it has no effect on the bacteria which lack complex V. Similarly, addition of the cyanide-4 (trifluoromethoxy) phenylhydrazone, an uncoupling agent that collapses the proton gradient, allows us to assess the spare respiratory capacity of the host cell, whether infected or not. We thus used these parameters to determine whether OxPhos was modified by infection in primary epithelial cells. We observed no significant difference in the mitochondrial-dependent OCR (Fig. 3*F*), nor in the spare respiratory capacity (Fig. 3*G*), in cells infected for 24, 30, or 48 h compared to control cells. Similar trends were seen with A2EN cells (Fig. 3, *H* and *I*). Thus, OxPhos is not altered by *C. trachomatis* infection.

### Glycolysis and OxPhos are both required for *C. trachomatis* proliferation in primary cells but their contribution to early development differ

We next investigated to what extent the bacteria require glycolysis and/or OxPhos to work at full capacity to undergo a normal developmental cycle by examining the consequence of chemical inhibition of these pathways on bacterial progeny 48 hpi.

**Figure 4:**
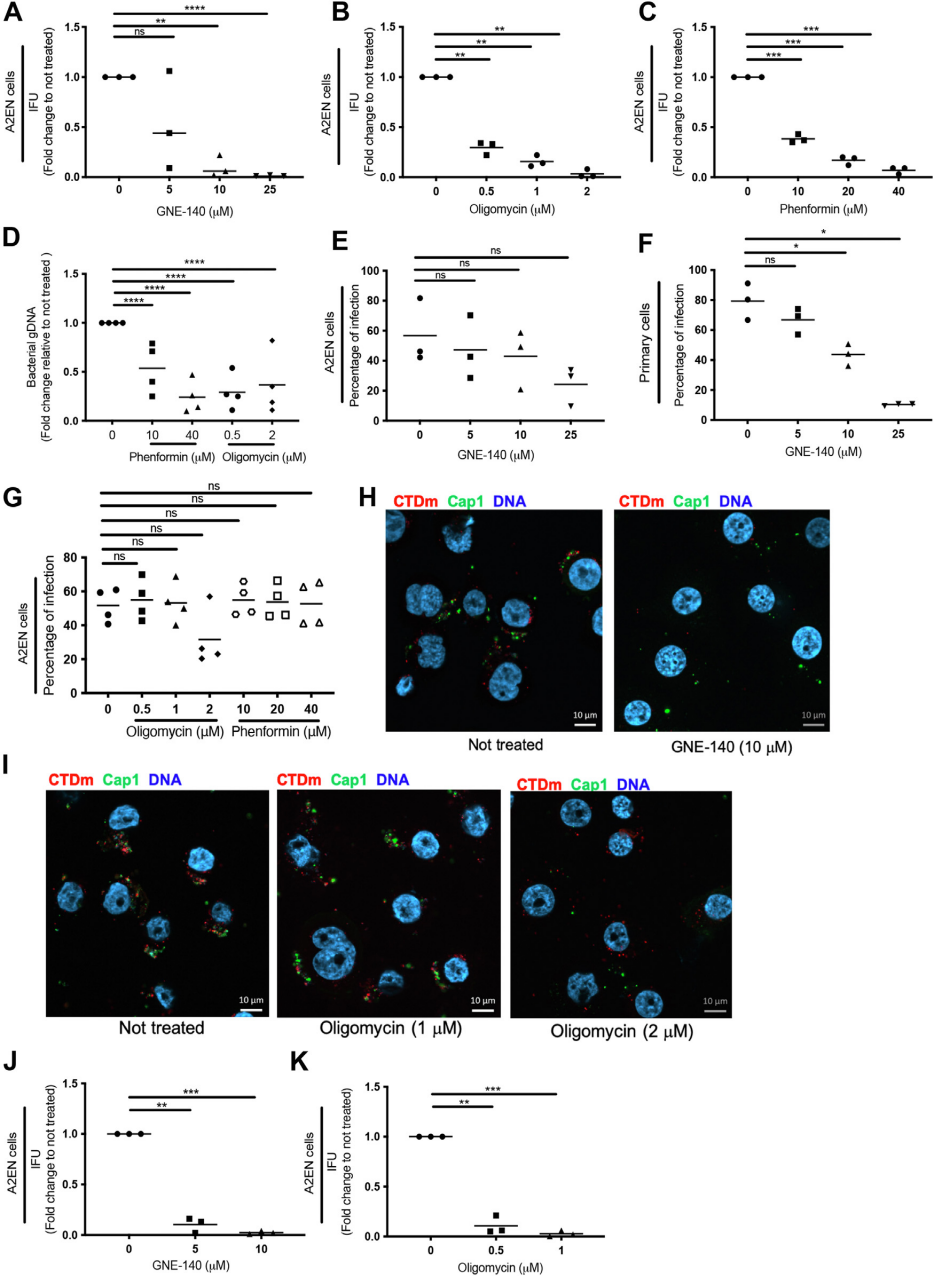
**Glycolysis and OxPhos are both required for bacterial proliferation in epithelial cells, glycolysis being also required for the initiation of infection.** A2EN cells were infected with CTDm and treated with indicated concentrations of GNE-140 (*A*), oligomycin (*B*), or phenformin (*C*) for 48 h. Cells were lysed and bacterial IFU were determined by reinfecting fresh HeLa cells as described in the Experimental procedures. *D*, A2EN cells were infected with CTDm and treated with indicated concentrations of oligomycin or phenformin for 48 h. Cells were lysed for DNA extraction and samples were analyzed by qPCR. Bacterial load was determined by quantifying the bacterial *glgA* gene and normalizing it to cell number using the *36B4* gene. A2EN (*E* and *G*) or primary cells (*F*) were infected for 24 h in the presence of the indicated concentration of drug before being fixed and analyzed by flow cytometry to determine the percentage of infected cells. A2EN cells were infected at MOI 20 with CTDm and treated or not with GNE-140 (*H*) or oligomycin (*I*). Cells were fixed 6 hpi, then permeabilized before immunostaining with anti-Cap1 followed with Alexa488-conjugated secondary antibodies. DNA is stained with Hoechst. *J* and *K*, HeLa cells were infected with CTDm and treated 12 hpi with indicated concentrations of GNE-140 (*J*) or oligomycin (*K*) before IFU were determined 48 hpi, as described in the Experimental procedures. The results of 3 independent experiments and the mean are displayed. For each panel, the results of 3 or 4 independent experiments and the mean are displayed. Significance is defined as: ns (not significant) *p* > 0.05; (∗) = *p* ≤ 0.05; (∗∗) = *p* ≤ 0.01; (∗∗∗) = *p* ≤ 0.001; (∗∗∗∗) = *p* ≤ 0.0001. CTD, *C. trachomatis* serovar D; hpi, hours postinfection; IFU, infection forming unit; OxPhos, oxidative phosphorylation; qPCR, quantitative PCR.

GNE-140 is a potent and specific LDH A/B inhibitor (23). Cells exposed to GNE-140 show an increase in metabolites associated with glycolysis and the pentose phosphate pathway, which lie upstream of LDH A/B, but levels of metabolites associated with OxPhos remain stable (23). In 2 different cancer cell line models, GNE-140 was shown to block the glycolytic pathway and force cells toward oxidative phosphorylation (24). We first tested the efficiency of GNE-140 by measuring its effect on lactate production, except for A2EN, whose lactate production was below the sensitivity threshold of the assay and for which ECAR measurement was used instead. Increasing doses of GNE-140 reduced both lactate production in HeLa cells (Fig. S4*A*) and ECAR in A2EN cells (Fig. S4*B*). Importantly, GNE-140 did not affect OCR in A2EN cells (Fig. S4*C*). It can be deduced from these observations that GNE-140 likely impairs glycolysis, but not OxPhos. Since the targets of GNE-140, LDH A/B, are absent in *C. trachomatis*, direct effect of the drug on the bacteria is unlikely. We thus decided to measure its effect on the completion of 1 developmental cycle. The progeny (IFU) collected after 48 h of infection in the absence or presence of the drug was measured by infecting HeLa cells and counting the percentage of infected cells 1 day later. We observed a dose-dependent reduction of the infectious progeny in A2EN cells treated with increasing doses of GNE-140, indicating that a functional glycolytic pathway in the host is required for bacterial development (Fig. 4*A*). Note that we did not observe important cell loss 48 hpi even at the highest concentration of drug used.

To examine whether bacterial development also required full OxPhos capacity of the host, we next measured the effect of the OxPhos inhibitor oligomycin on IFU formation. As an alternative drug, we used phenformin, an inhibitor of the complex I of the mitochondrial respiratory chain (25). We observed a dose-dependent reduction of the progeny for both drugs. For instance, the number of infectious particles collected from A2EN 48 hpi was reduced by 60 to 75 % when the culture had been done in the presence of 0.5 μM oligomycin (Fig. 4*B*) or 10 μM phenformin (Fig. 4*C*). To directly measure the effect of these drugs on bacterial proliferation, we used qPCR to estimate the relative number of bacterial genomes in the different culture conditions. We observed a similar decrease in the number of genomes as in the number of IFUs, indicating that the drop in IFU when OxPhos was impaired was reflecting a decrease in bacterial numbers at the previous infection round (Fig. 4*D*).

These data show both glycolysis and OxPhos are required for optimal bacterial growth and that 1 pathway cannot compensate for the other. However, when measuring the percentage of cells infected in the presence of inhibitors, differences between the 2 pathways appeared. We observed a trend toward a diminution of the percentage of infected A2EN cells with increasing concentrations of GNE-140, a finding that we confirmed in primary cells (Fig. 4, *E* and *F*). In contrast, 40 μM phenformin or 1 μM oligomycin, which, like 10 μM GNE-140, diminished IFUs by more than 80% (Fig. 4, *A–C*), did not affect the percentage of infected cells (Fig. 4*G*). The decrease in the percentage of infection upon GNE-140 treatment suggested that establishment of the infection might be impaired. To test this hypothesis, we examined cells 6 hpi, a time point at which the bacteria are expected to have clustered at the microtubule organizing center (MTOC) (26). As a readout of the establishment of the early inclusion, we used an antibody against Cap1. Cap1 is one of the bacterial proteins secreted early in the inclusion membrane (27). Six hours postinfection, GNE-140 treatment resulted in a decrease in bacterial clustering near the nucleus (Fig. 4*H*). Cap1-positive staining was observed in both nontreated and GNE-140–treated cells, but appeared reduced in the drug-treated cells, possibly due to the clustering defect, which dispersed the signal throughout the cell volume. In contrast, 1 μM oligomycin did not seem to affect early infection, and it is only when used at 2 μM concentration that a defect in bacterial clustering was noticed (Fig. 4*I*). These observations, together with the effect of drug treatment on the percentage of infected cells, indicate that early bacterial development is more sensitive to GNE-140 treatment than to OxPhos inhibition. Finally, to determine if the impairment of the early steps of bacterial development accounted for the overall effect of GNE-140 treatment in progeny assays, we delayed the treatment to 12 hpi, thus after the completion of the initial steps of infection (Fig. 4*J*). If anything, we observed a stronger effect of GNE-140 when applied 12 hpi *versus* at the onset of infection (compare Fig. 4, *A* and *J*). Oligomycin also had a more potent effect when applied 12 hpi than 2 hpi (Fig. 4*K*). These data show that glycolysis is not only required at the onset of the developmental cycle but also for bacterial proliferation. During this phase of development, bacteria require both glycolysis and OxPhos to run at their full capacity for the bacteria to reach optimal growth.

### The bacterial reliance on the glycolytic pathway of the host is by-passed in 2 cancer-derived cell lines

Surprisingly, in contrast to what we observed in primary cells, in HeLa cells, bacterial development was largely insensitive to GNE-140, even at concentrations that completely block lactate production (Figs. 5*A* and S4*A*). Consistent with this observation, the early steps of infection appear to proceed normally in HeLa cells in the presence of GNE-140 (Fig. 5*B*). This result is important as it rules out the possibility that the drug impaired bacterial growth in primary cells *via* direct effect on *C. trachomatis*. Furthermore, this suggests that the requirement for full capacity glycolysis by *C. trachomatis* is by-passed in the HeLa cell line. Indeed, silencing glucose phosphate isomerase (GPI), one of the early enzymes that control the flux into the glycolytic pathway, had no effect on progeny in HeLa cells (Fig. 5*C*). As control of the efficiency of the silencing strategy at blocking glycolysis, we observed that lactate production was decreased by 75% by the 2 siRNAs used (Fig. 5*D*). To test another cell line, we used an epithelial cell line derived from the human colon, LS174T, in which *GPI* has been knocked-out. These KO cells have been well characterized and rely only on OxPhos for growth (28). When IFUs were titrated 48 hpi on 2 independent *GPI* KO clones, no difference was found compared to IFUs obtained from infecting the parental WT LS174T cells (Fig. 5*E*). Thus, the reliance of *C. trachomatis* on the glycolytic pathway was observed in A2EN but not in 2 cell lines of tumor origin.

### The metabolic autonomy of EBs is insufficient to fully complete the initial steps of infection

**Figure 6:**
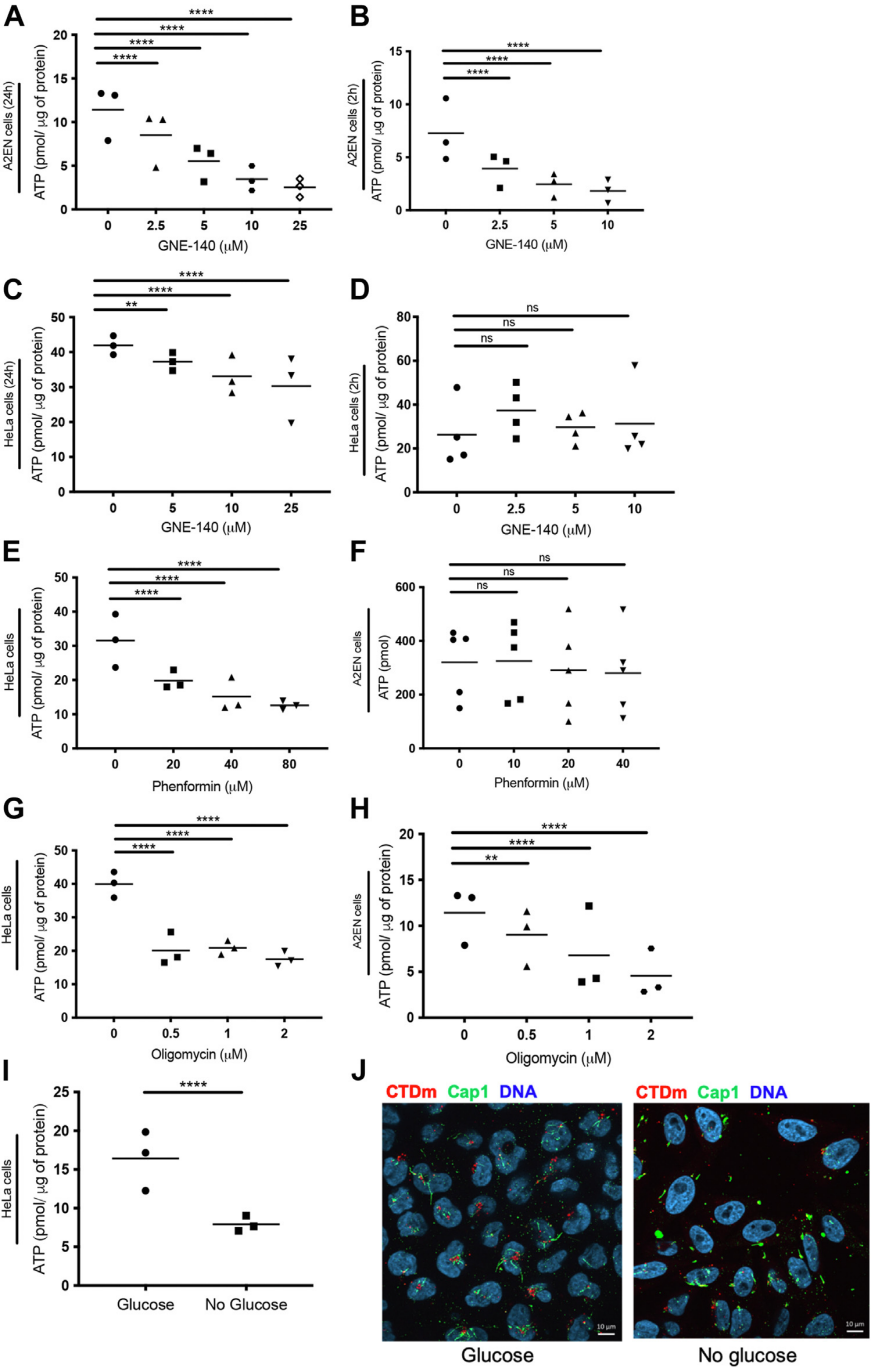
**ATP levels are differentially affected by GNE-140 and OxPhos inhibitors in primary and HeLa cells.** A2EN cells (*A*, *B*, *F*, and *H*) and HeLa cells (*C*, *D*, *E*, and *G*) were treated with the indicated concentrations of GNE-140, oligomycin, or phenformin for 24 h except in (*B*) and (*D*) where cells were treated for 2 h. ATP levels were measured as described in the Experimental procedures. *I*, HeLa cells were grown in medium containing glucose or not for 48 h. Intracellular ATP levels were then measured as described in the Experimental procedures. *J*, HeLa cells were grown in medium containing glucose or not for 48 h before infection with CTDm at MOI 10. Cells were fixed 6 hpi, then permeabilized before immunostaining with anti-Cap1 followed with Alexa488-conjugated secondary antibodies. DNA is stained with Hoechst. For each panel, the result of 3 independent experiments and the mean are displayed. Significance is defined as: ns (not significant) *p* > 0.05; (∗∗) = *p* ≤ 0.01; (∗∗∗∗) = *p* ≤ 0.0001. CTD, *C. trachomatis* serovar D; hpi, hours postinfection; OxPhos, oxidative phosphorylation.

The lack of effect of glycolysis inhibition on bacterial progeny in HeLa cells, when it clearly affected several steps of bacterial development in A2EN cells and primary cells, was puzzling. Cancer lines often have a strong basal glycolytic rate. We thus expected inhibition of this pathway to have a stronger impact on ATP levels, and thereby on bacterial development, in HeLa cells than in A2EN cells. To understand the respective contribution of these pathways at maintaining ATP balance, we first compared the effect of GNE-140 on ATP levels in A2EN and in HeLa cells. In A2EN cells, application of GNE-140 for 24 h induced a dose-dependent decrease in intracellular ATP (Fig. 6*A*). The effect of the drug was actually very fast, since it was significant after only 2 h of treatment for 2.5 μM GNE-140 (Fig. 6*B*). In contrast, in HeLa cells, 2 h treatment with 10 μM GNE-140 had no significant effect on ATP levels, and for long term treatment (24 h), the drop in ATP level was only of about 30% for 25 μM GNE-140 (Fig. 6, *C* and *D*). Consistent with OxPhos being more important to maintain ATP levels in HeLa cells than in A2EN, we found that phenformin treatment applied for 24 h affected ATP balance in HeLa cells and not in A2EN (Fig. 6, *E* and *F*). The 2 cell lines showed similar sensitivity to 24 h oligomycin treatment (Fig. 6, *G* and *H*). Note than phenformin and low concentrations of oligomycin had no significant effect on ATP levels in HeLa cells when applied for 2 h only (Fig. 5, *A* and *C*). For this short exposure time, the OxPhos inhibitors also had only marginal (<25% decrease) effects on ATP level in A2EN (Fig. 5, *B* and *D*).

Since GNE-140 treatment resulted in a rapid drop in ATP levels in primary cells, and not in HeLa cells, we wondered if this observation could explain the difference of sensitivity to GNE-140 of the early steps on infection depending on the cellular background. Indeed, the presence of glycogen and of glycolytic enzymes in EBs might cover the intrinsic energetic needs of the bacteria in the first hours of the infectious cycle, so that access to external glucose source might not be required. However, migration of the bacteria to the MTOC and extension of the inclusion membrane are host-driven processes that consume ATP, thus requiring a normal ATP balance in the host. To challenge this hypothesis, we removed glucose from the culture medium of HeLa cells, thus reducing ATP production by both glycolysis and OxPhos. Indeed, the cellular ATP level measured 48 h after the initiation of glucose deprivation was decreased by 50% (Fig. 6*I*). To determine the impact of this on infection, HeLa cells grown in the presence or absence of glucose were infected with CTDm, fixed 6 hpi, and stained for the inclusion protein Cap1. In cells cultivated in the presence of glucose, many of the bacteria were clustered near the nucleus, and Cap1-positive tubules were detected around them. In contrast, in glucose-deprived cells, bacteria were less clustered (Fig. 6*J*). Cap1 staining was detected, but the tubules were shorter than in control cells. Their observations are similar to what we observed in A2EN treated with 10 μM GNE-140 (Fig. 3*H*), with the difference that Cap1-positive tubules are thinner and longer in HeLa than in A2EN. Thus, although EBs do not need external source of glucose to initiate the transcription and translation of early genes like *cap1*, completion of some of the early steps of infection, for example, clustering to the MTOC and inclusion tubule extension, required glucose to be present in the culture medium. Furthermore, failure to complete the initial steps of infection in the 2 cellular backgrounds correlated with low cellular ATP levels, supporting the hypothesis that the effect of GNE-140 treatment on the early step of infection in A2EN cells and primary cells is due to limited ATP availability.

## Discussion

In this study, we measured the consequences of an infection by an intracellular organism on the 2 main sources of ATP in primary cells. We observed that OxPhos remained stable. Glycolysis increased only slightly, mostly at the end of the developmental cycle. These observations suggest that the metabolic pressure exerted by the bacteria remains within the limit of what the host cell metabolic buffering capacity without strongly enhancing host ATP production to compensate for infection. However, application of inhibitors of glycolysis or of oxidative phosphorylation reduced the formation of infectious bacteria in a dose-dependent manner, indicating that both pathways are required to function at full capacity to sustain bacterial growth. Finally, although, as expected, EBs displayed some metabolic autonomy, a functional glycolysis in the host was needed even at the onset of the infectious process.

This represents the first comprehensive study of the consequence of *C. trachomatis* infection on primary epithelial cells. The difficulty to obtain patient-derived cells led us to also use 2 cervical, nontumor–derived, epithelial cell line, A2EN and End1/E6E7. For all experiments in which we could compare the metabolism of these cell lines and primary cells, we observed similar phenotypes, including in the context of *C. trachomatis* infection. A2EN and End1/E6E7 are therefore a suitable surrogate to primary cells to study metabolic flux, and we will refer to the generic term “primary cells” in the rest of the discussion.

This study was conducted with a serovar of *C. trachomatis* more representative of the circulating sexually transmitted infections than the more commonly used L2 serovar. For practical reasons, we generated a strain stably transformed with a *C. trachomatis* L2-derived plasmid engineered to constitutively express the fluorescent protein mCherry. As previously shown for GFP-expressing *C. trachomatis* L2, it enables to easily quantify infected cells by flow cytometry. However, unlike GFP (29), the mCherry signal was slightly too weak for reliable detection by immunofluorescence on single EBs. Interestingly, we observed that the fluorescent bacteria were more infectious than the parental ones, due to enhanced attachment and intracellular growth. This is in contrast to stably transformed serovar L2 strains that did not differ from the parental strain for these 2 parameters (29). To the best of our knowledge, this is the first detailed investigation of the difference *in vitro* between a plasmid-bearing parental strain and the transformed strain for a non-LGV strain. In the LGV serovars of *C. trachomatis*, in which transformation protocols were initially implemented, comparison of parental and transformed strains led to the conclusion that there was no significant difference in infectivity (17, 30). Importantly, LGV strains are more infectious than the ocular or other genital serovars *in vitro*. It might be that the advantage provided by the transformation is not significant in the LGV background that proliferates already extremely well in tissue culture.

We measured that the CTDm contained an average 3-fold more copies of the plasmid than the parental bacteria. The chlamydial plasmid was shown to provide a selection advantage over plasmid-free bacteria *in vitro* and *in vivo* (18). In particular, Pgp3, 1 of the 8 proteins coded by this plasmid, is required for full virulence *in vitro* and *in vivo* (31) and might be exposed on the EB surface (32). Thus, increase in Pgp3 expression might account for the enhanced infectivity observed with the fluorescent strain. Altogether, we concluded from these preliminary experiments that the fluorescent bacteria might exert a metabolic pressure slightly earlier than the parental strain and make a suitable and convenient tool to study the intracellular development of *C. trachomatis* serovar D.

ATP levels remained stable over the length of CTD infection in primary cells. In A2EN cells, we did not detect an increase in phosphorylation of ACC, indicating that cytosolic ATP level was preserved in this cellular background. In contrast, we observed a drop in ATP levels in infected HeLa cells, which was mirrored by a drop in cytosolic and mitochondrial ATP measured using fluorescent probes. We did not observe an increase in ATP level in HeLa cells 8 hpi, unlike what was reported in a previous study using the LGV serovar (20). Also, at odds with our observations, mitochondrial activity was reported to increase in HeLa cells infected for 24 h with CTD (33). The main difference between these studies and ours are the cellular backgrounds and the strains used. As discussed further below, our results provide several illustrations of the difference in the basal metabolism of HeLa cells and primary epithelial cells, and it is likely that infection has different effects on the metabolism of these 2 cell types.

Based on *C. trachomatis*’ capacity to hijack ATP from its host, and the increase in glucose uptake by infected cells, we expected to observe a shift in at least 1 of the 2 main ATP generating pathways in host cells, that is, glycolysis and OxPhos. Surprisingly, we observed only a moderate increase in glycolysis, mostly at the end of the bacterial developmental cycle, and no change in OxPhos. The measures of ECAR in A2EN cells indicated a small increase in glycolysis in the second half of the infectious cycle. This was confirmed by measuring lactate production in End1/E6E7 cells. Increase in lactate production was also observed in HeLa cells 48 hpi, in agreement with published data (14). Interestingly, the increase in lactate production 48 hpi in End1/E6E7 cells became significant after normalizing to cell number, due to a drop in cell numbers in the infected wells compared to their noninfected counterpart (compare Fig. 3, *D* and *E*). The drop in cell number over the course of infection is likely due to the well-documented decrease in cell division in cells infected by *C. trachomatis* (34, 35), although some cell death might also occur. Thus, overall, it appears that the infected cells maintain their level of glycolysis, in spite of reducing their own growth. This was also observed in HeLa cells. Interestingly, it was reported that protein synthesis also decreases in infected cells (36). Decrease in protein synthesis and cell division during infection might contribute to maintaining ATP levels in the host, in spite of bacteria hijacking part of the host production.

Overall, we concluded from our observations that infection does not induce a major shift in the energy metabolism of the host primary cells, suggesting that their basic metabolism is sufficient to cope with bacterial needs. In fact, glucose is known to be hijacked directly by the bacteria and captured in the form of glycogen in the inclusion, of hexosamines, and of cell wall components (3, 5, 37). There might simply not be sufficient glucose to boost ATP synthesis in the host, leaving glycolysis and OxPhos largely untouched.

This does not mean that these 2 pathways are not required for bacterial growth. In fact, we observed that even low concentrations of chemical inhibitors of either pathway induced a decrease in bacterial progeny measured 48 hpi in primary cells, implicating that both pathways are required to function a full capacity for optimal *C. trachomatis* growth. In A2EN cells, we had no other mean of intervention on glycolysis than the use of the chemical inhibitor GNE-140, as other “glycolysis inhibitors” have pleiotropic effects, and gene silencing or disruption did not work in this cell line. We showed that 10 μM GNE-140 induced a rapid and significant drop in ATP levels in A2EN without affecting OxPhos, implicating that it blocks glycolysis-driven ATP production. GNE-140 affects a step downstream of glycolysis, meaning that glycolytic intermediates may still be present and even increased in GNE-140–treated cells, as was shown in a pancreatic cell line (23). Our data indicate that glycolysis is required to sustain the proliferative stage of the development. Indeed, inhibition of LDH A/B, either from the beginning or during mid-stages of infection, severely reduced bacterial progeny measured 48 hpi. Importantly, treatments with 10 μM GNE-140 had no effect on bacterial progeny in HeLa cells, ruling out the possibility that the drug is acting directly on the bacteria. Considering the strong effect of GNE-140 on ATP levels in primary cells, it is likely that GNE-140 delays bacterial replication by limiting bacterial access to host ATP. Nevertheless, we cannot exclude the possibility that the effect of GNE-140 treatment is linked to other metabolic changes. On 1 hand, GNE-140 treatment induces an accumulation of some intermediates of the pentose phosphate pathway, which could be utilized for bacterial nucleotides synthesis (23). GNE-140 treatment also decreases NAD^+^/NADH ratio, with both a decrease in NAD^+^ and an increase of NADH, which could be detrimental to bacterial development, as discussed below. Furthermore, a recent study demonstrated that 3 glycolytic enzymes, aldolase A, pyruvate kinase, and LDH, were enriched at the inclusion membrane, and the former was proven to be important for inclusion formation and bacterial development. The authors suggested that recruitment of host glycolytic enzymes at the inclusion membrane could be a way by which the bacteria have easier access to ATP or small metabolites produced through the glycolytic pathways or to nucleotides produced by the pentose phosphate pathway (15). GNE-140 treatment might affect the localization of these enzymes, so that even if the intermediates of glycolysis are still being made in the presence of the drug, they might have lost in accessibility for the bacteria. Interestingly, inhibition of the glycolytic pathway in 2 cell lines of tumor origin, HeLa cells and LS174T cells, using either gene silencing or gene disruption, had no impact on CTDm progeny. Inhibition of LDH A/B had also no effect in HeLa cells, while it had a strong effect on progeny in primary cells. These observations emphasize the need for primary cells to study metabolic requirements.

Our data show that OxPhos is also required for bacterial growth in primary epithelial cells, as 2 distinct OxPhos inhibitors decreased IFU production in a dose-dependent manner. Tipples and McClarty showed *C. trachomatis* grew normally in a cell line deficient of mitochondrial function, indicating that mitochondrial ATP might be dispensable (38). A more recent work in HeLa cells showed that infection preserves the mitochondrial network, and that mitochondrial ATP is required for bacterial development (39). Reduction of inclusion size upon oligomycin treatment in HeLa cells was also reported (22). Together with our own observations in primary cells, we can conclude that OxPhos needs to function at full capacity for optimal *C. trachomatis* growth. The bacterial need for host ATP might account for the effect of the drugs on bacterial proliferation. However, the comparison of the effect of GNE-140 and oligomycin on progeny and on ATP level call for additional explanations. Indeed, when applied 12 hpi, 0.5 μM oligomycin or 5 μM GNE reduced IFUs by 90 to 95%. However, 0.5 μM oligomycin reduced ATP levels in 24 h by only 20%, when 5 μM GNE did so by 50%. One metabolic imbalance caused by blocking OxPhos likely to affect bacterial growth is the decrease in NAD regeneration (40). *C. trachomatis* uses its ATP/ADP transporter, Ntp1, for NAD transport (41, 42). Reduction in NAD import from the host is expected to limit oxidative phosphorylation in the bacteria, thereby affecting their growth. Imbalance of other metabolites in the host because of OxPhos inhibition might also contribute to the strong decrease in IFUs observed.

Glycogen synthesis genes are present in all known families of *Chlamydiae*, including *Estrella lausannensis* and *Waddlia chondrophila*, which use a very peculiar pathway for its synthesis (43). This observation is surprising since glycogen metabolism loss appears to be universal to most if not all obligate intracellular bacterial pathogens or symbionts that went through genome reduction throughout evolution (44). This strongly suggests that glycogen plays an essential role in the developmental cycle of *Chlamydia*e. In *C. trachomatis*, proteomics studies have shown a very strong enrichment for enzymes involved in glycogen synthesis and degradation in EBs compared to RBs, and glycogen is only present in EBs (3, 4). These observations support the hypothesis that EBs, more than RBs, uses glycogen to sustain their metabolic need. Catabolism of intrabacterial glycogen into G1P, and conversion to G6P with a phosphoglucomutase, may feed the glycolysis pathway and generate ATP during the extracellular stage and in the very early stages of intracellular development, when import of host ATP has not been established (45). We hypothesized that EBs might have some degree of autonomy and that early steps of infection might occur normally even if glycolysis or oxidative phosphorylation were not functioning at full capacity. However, GNE-140 treatment reduced the capacity of the bacteria to establish an infection in primary cells, with a defect in bacteria clustering near the nucleus, and in the extension of inclusion tubules. Interestingly, by changing HeLa cell metabolic state though deprivation of glucose, a similar phenotypic growth defect was observed. Motor-driven transport of bacteria along microtubules and fusion with host compartments for building the inclusion membrane are host-driven processes that require energy. It is thus likely that the sensibility of the early steps of development in the 2 cellular backgrounds, either by GNE-140 treatment or by glucose deprivation, is related to the drop of ATP we observed in both cases. We concluded from these observations that EBs exhibit some degree of metabolic autonomy but rely on energy-driven processes of the host to establish the nascent inclusion.

Finally, few studies investigating the metabolic requirements of intracellular bacteria in epithelial cells have been completed to date. A majority of our knowledge on how host metabolism responds to infection has been obtained from macrophages, an innate immune cell specialized for bacterial clearance. In these phagocytic cells, several studies have shown that infection commonly upregulates glycolysis, a phenomenon required to mount the host inflammatory response to infection (46). The difficulty to obtain primary epithelial cells probably explains this gap. Interestingly, a pioneering study by the Bumann lab showed cells infected by *Shigella flexneri* maintained largely normal fluxes through glycolytic pathways and preserved host cell ATP generation, despite vigorous exploitation of the metabolites of the host by the bacteria (47). These conclusions are comparable to our own with *C. trachomatis* infection of epithelial cells. More studies on infection in epithelial cells are however necessary to generalize this picture, starting with other *Chlamydiaceae*. Indeed, *Chlamydia pneumoniae* growth is promoted in hypoxic conditions, that is, when mitochondrial functions are impaired, while hypoxia had no effect on *C. trachomatis* progeny in primary cells derived from Fallopian tubes (48, 49). Furthermore, silencing the ATP synthase promoted *C. pneumoniae* growth in both normoxia and hypoxia (48), while we observed here that OxPhos is essential for optimal growth of *C. trachomatis*. Thus, the fine metabolic equilibrium reached by *C. trachomatis* and epithelial cells of the genital tract might be achieved by different means for other *Chlamydia* species and might play a role in its tissue tropism.

## Experimental procedures

### Cells and bacteria

Human cervix–derived epithelial HeLa cells (ATCC), human colon–derived epithelial LS174T WT and GPI-KO cells (28) were grown in Dulbecco’s modified Eagle’s medium with Glutamax (DMEM, Thermo Fisher Scientific), supplemented with 10 % (v/v) heat-inactivated fetal bovine serum (complete medium). LS174T cells were a kind gift from Dr J. Pouysségur (Institute for Research on Cancer and Aging). Primary epithelial cells were isolated from ectocervix biopsies of female patients after approval by the French Ethical Committee ‘CPP Ile de France 1’ on May 9, 2016. Approval and authorization of the National Data Protection authority (‘Commission Nationale de l’Informatique et des Libertés’, CNIL) have been obtained for the research protocol, in compliance with the Helsinki principles. Primary cells were cultivated in keratinocyte serum free medium (Thermo Fisher Scientific), containing 50 mg/l of bovine pituitary extract (Thermo Fisher Scientific) and 5 μg/l of epidermal growth factor human recombinant (Thermo Fisher Scientific) (50). They were used between passage 3 and 6. The endocervical cell lines End1/E6E7 (ATCC) and A2EN (kind gift from Dr Alison Quayle) were cultivated in the same conditions as the primary epithelial cells and used at passage 9 to 30. *C. trachomatis* serovar D/UW-3/CX (CTD) and mCherry transformed serovar D/UW-3/CX (CTDm) were propagated in HeLa cells, as described (51). EBs were stored in sucrose-phosphate-glutamic acid buffer (10 mM sodium phosphate [8 mM Na2HPO4-2 mM NaH2PO4], 220 mM sucrose, 0.50 mM l-glutamic acid) at −80 °C. The L2-based p2TK2-SW2 plasmid was a kind gift of Dr Derré (University of Virginia). It encodes the fluorescent protein mCherry under the control of the promoter of the early gene *incD* (17) and was used to transform CTD following standard procedures (30).

### Cell culture and treatment

Cells were cultured at 37 °C, in 5 % CO2 atmosphere and were routinely tested, with bacteria stocks, for *mycoplasma* using the standard PCR method. Confluent cultures of A2EN and primary cells were detached with 0.3% trypsin/EDTA for 3 min at 37 °C before a wash in RPMI 1640 (Thermo Fisher Scientific). Cells were centrifuged for 15 min at 2500×*g* and the pellet was resuspended in keratinocyte serum free medium to be seeded or passed. When confluent, HeLa cells and LS174T cells were detached with 0.5 mM EDTA in PBS before being seeded or passed in complete medium. For experiments of glucose deprivation from HeLa cells, cells were seeded in normal medium. On the following day, the medium was replaced with DMEM without glucose (Thermo Fisher Scientific, A14430) complemented with 5 mM sodium pyruvate (Sigma-Aldrich), 10% FBS. Experiments were performed 48 h from the start of glucose deprivation. Experiments preceding lactate and ATP measurements in HeLa cells were performed in DMEM medium deprived of pyruvate (Thermo Fisher Scientific, 61965059). CTD and CTDm infections were always followed by a 30 min centrifugation step at 270×*g* at 37 °C, unless otherwise stated. For the different durations of infection that we analyzed, infections were initiated sequentially, so that all samples were collected and analyzed together. Stock solutions of the chemical used were as follows: phenformin, 200 mM in H2O (Sigma-Aldrich, P7045); Oligomycin, 5 mM in DMSO (Cayman Chemical, 11341); GNE-140, 4 mM in DMSO (Sigma-Aldrich, SML2580). All chemicals were stored at −20 °C.

### Adhesion assay

Adhesion assays were performed as previously described (Vromman *et al.*, 2014). HeLa cells were plated in a 24-well plate (10^5^ cells/well) the day before the assay. Cells were precooled 30 min at 4 °C after what they were incubated for 4 h at 4 °C (including a 30 min centrifugation step also at 4 °C) with either equal genome number of CTD or CTDm. Prior to infection, bacteria were gently sonicated (3 times 5 s) in order to disrupt bacterial aggregates. Cells were then washed gently with cold PBS, lysed directly in the plate followed by genomic DNA (gDNA) extraction using the DNeasy Blood and Tissue Kit (Qiagen). Bacterial adhesion was determined through qPCR.

### Progeny assay and flow cytometry

Cells were seeded in a 24-well plate the day prior to infection. For experiments using chemical inhibitors, GNE-140, oligomycin, or phenformin were added to the culture medium at the indicated concentrations when infecting cells, unless otherwise stated. Cells with an infection lower than 30 % (checked by microscopy, and at least 50% for A2EN cells) were detached 48 hpi (or 30 hpi for the silencing experiment) with 0.5 mM EDTA in PBS for HeLa cells or 0.3 % trypsin for LS174T cells, primary cells, and A2EN cells and analyzed by flow cytometry to determine the bacterial titer (29). Cells infected using CTDm were analyzed directly on 1/10 of the sample diluted in PBS. Cells infected with CTD were stained with a mouse antibody against Hsp60 (MA3-023, Thermo Fisher Scientific) followed with Alexa488-coupled secondary antibody against mouse (Molecular Probes). Flow cytometry analysis was performed with a CytoFLEX S (Beckton Coulter). A minimum total of 10,000 gated events were collected for each sample. Data were then analyzed using FlowJo software (version 10.0.7) to determine the bacterial titer as described in (Vromman *et al.*, 2014).

### Immunofluorescence

All cells were grown on coverslips in a 24-well plate. After the indicated time of infection, the cells were fixed in 4% paraformaldehyde (Merk-Millipore) 4% sucrose in PBS for 20 min and was followed with 10 min quenching with 50 mM NH4Cl in PBS. Cells were permeabilizing in 0.05% Saponin (Sigma-Aldrich), 1 mg/ml bovine serum albumin (Sigma-Aldrich) in PBS for 10 min. Primary antibodies used in this study were anti-*Chlamydia* antibody (a home-made mixture of rabbit polyclonal antibodies against several *Chlamydia* antigens), rabbit anti-phospho-Acetyl-CoA Carboxylase (Ser79) (Cell Signaling #3661), and rabbit anti-Cap1 antibody (3). DNA was stained using 0.5 μg/ml of Hoechst 33342 (Thermo Fisher Scientific) which was added to the secondary antibody. Coverslips were then mounted in Mowiol (Sigma-Aldrich). Images were acquired on an Axio observer Z1 microscope equipped with an ApoTome module (Zeiss) and a 63 × Apochromat lens. Images were taken with an ORCAflash4.OLT camera (Hamamatsu) using the Zen software. Images were analyzed with ImageJ software. CTD and CTDm inclusions size was determined using the cell image analysis software CellProfiler.

### qPCR

Total DNA was isolated with the DNeasy Blood and Tissue Kit (Qiagen). To determine plasmid number and bacterial genome number (gDNA), 100 μl of CTD and CTDm EBs were used for DNA extraction. DNA concentrations were determined with a spectrophotometer NanoDrop (Thermo Fisher Scientific) and normalized to equal contents. qPCR was performed using LightCycler 480 SYBR Green Master I (Roche) following the manufacturer’s instructions. To normalize for equal genome number, a standard curve was made using a plasmid containing the target gene *glgA* used to measure genome number as described in the Applied Biosystem protocol “Creating Standard Curves with Genomic DNA or Plasmid DNA Templates for use in quantitative PCR” (2003). Primers against a conserved sequence within ORF2 were used to assess plasmid number and then were analyzed using the ΔΔCt method (52). To determine gDNA quantities relative to cell (adhesion assay, assessment of bacterial genomes in cells infected in the presence of oligomycin), primers for *glgA* and for the 36B4 gene (encoding for an acidic ribosomal human phosphoprotein P0) were used to determine the relative number of bacteria per cell. Each qPCR experiment was performed in triplicate and repeated at least 3 times. The primer sequences are as follows: *glgA* Forward: 5′-AATGATTGGAATGCGTTACGG-3′, Reverse: 5′-CGGTAGGTTGTCACTGCTTCC-3′; 36B4 Forward: 5′-TGCATCAGTACCCCATTCTATCAT-3′; 36B4 Reverse: 5′-AAGGTGTAATCCGTCTCCACAGA-3′; Ctrachplas Forward: 5′-CAGCTTGTAGTCCTGCTTGAGAGA-3′; Ctrachplas Reverse: 5′-CAAGAGTACATCGGTCAACGAAGA-3′.

### Measurement and quantification of intracellular ATP

Intracellular levels of ATP were analyzed using the CellTiter Glow luminescence assay (Promega) according to the manufacturer's instructions. Cells (60 × 10^3^ for HeLa in a 48-well plate, 75 × 10^3^ for primary cells in a 48-well plate, and 30 × 10^3^ for A2EN cells in a 96-well plate) were treated or infected for the indicated time in pyruvate-free medium, so that more than 75% of the cells were infected on the following day (checked under the microscope). The cell medium was then discarded or used for lactate measurement in the case of primary cells. Cells were then lysed directly in the plate with ATP Assay Buffer after what luminescence was measured with the Cytation 5 Cell Imaging Multi-Mode Reader (BioTek). ATP concentrations were calculated using an ATP (Sigma-Aldrich) standard curve (0–200 pmole). To normalize ATP levels to protein levels, duplicate wells of cells were lysed in 2 M urea buffer (Tris 30 mM, NaCl 150 mM, 2 M urea, 1 % SDS, pH=8.0) after what a BCA assay kit (Pierce) was used as indicated by the manufacturer to measure proteins levels.

### FLIM microscopy

The GO-ATEAM2 and mitoGO-ATEAM2 vectors suitable for expression in mammalian cells were previously described (21) and were a kind gift of Hiromi Imamura (Kyoto University). The mitoGFP-ATEAM2 donor-only control used to normalize the mitoGO-ATEAM2 lifetime values was obtained by subcloning the *Bacillus subtilis* ε subunit fused to cp173 mEGFP after digesting the original mitoGO-ATEAM2 vector with BshT1/HindIII. The cloning reaction was performed with the NEBuilder HiFi DNA Assembly Mix (New England Biolabs) and verified on a 3130 XL sequencer (Applied Biosystems). All restriction enzymes were purchased from Thermo Fisher Scientific. FLIM analyses were performed in the time domain, as described in (53). The FastFLIM setup used for FLIM acquisitions was driven by the Inscoper hardware (Inscoper). Cells were excited at 480 ± 10 nm using a white light laser, and emission was selected using a band-pass filter of 525/50 nm. GFP was used as a FRET donor in all experiments, its decrease in fluorescence lifetime was measured with the FLIM microscopy Suite (Inscoper). To calculate FRET variations using FLIM, the mean lifetime of cells expressing a donor-only version of the mito-ATEAM 2.0 (mitoGFP-ATEAM2) biosensor was first calculated for each condition. mitoGFP-ATEAM2 mean lifetime values were then used to normalize data in each experimental condition where the mitoGO-ATEAM2 biosensor was expressed. Last, ΔLifetime values were obtained by normalizing each experimental condition to the corresponding “Not infected” 1 for both mitoGO-ATEAM2 and GO-ATEAM2 vectors.

### Metabolic flux analysis

Seahorse XF96 extracellular flux analyzer (Agilent Technologies) was used to measure OCRs and ECARs following the Mito Stress test and Glyco stress test protocols, respectively (54). Cells were seeded (25,000 cells/well) on Seahorse 96-well plate and were infected at various times, so that on the day of measurement, cells had been infected (>75% infected cells) for 24, 30, or 48h or left uninfected. One hour prior to the assay, cell media was replaced by Seahorse XF Base Medium (Agilent Technologies), 1 mM glutamine (Sigma-Aldrich) adjusted at pH 7.4 for the Glyco Stress test. For the Mito Stress test, the medium was replaced with Seahorse XF Base Medium (Agilent Technologies), 1 mM pyruvate (Sigma-Aldrich), 2 mM glutamine (Sigma-Aldrich), and 10 mM glucose (Sigma-Aldrich), pH 7.4), and the plates were incubated in a non-CO2 incubator at 37 °C. For both tests, basal levels of OCR and ECAR were recorded for 30 min before consecutive addition of drugs that modulate OxPhos or glycolysis. In the Mito stress test, we added 2 μM oligomycin, 1 μM Carbonyl cyanide-4 (trifluoromethoxy) phenylhydrazone (Cayman Chemicals, 10 mM stock in DMSO), and a mix of 0.5 μM rotenone (Sigma-Aldrich, 10 mM stock in DMSO) and 0.5 μM antimycin A (Sigma-Aldrich, 10 mM stock in ethanol). In the Glyco Stress test, we added 10 mM glucose (Sigma-Aldrich), 2 μM oligomycin, 50 mM 2-Deoxy-D-glucose (Sigma-Aldrich). To normalize the data to cell number, plates were fixed at the end of the measurements in 2.5% paraformaldehyde for 10 min, then stained in 5 μg/ml DAPI (Invitrogen #D1306), 3% bovine serum albumin in PBS. They were then washed 3 times with PBS then before analysis by a Cytation 5 Cell Imaging Multi-Mode Reader (BioTek) for cell count. Images were acquired on a 7 × 7 grid (394 μm × 291 μm for each square) and were analyzed with the cell image analysis software CellProfiler. In summary, the pipeline counted DAPI-stained nuclei and subtracted mCherry-stained inclusions from the nuclei to give the cell count per well. The obtained count was adjusted according to the actual number of pictures obtained per well and the size of the Seahorse 96-well plate well (0,106 cm^2^). Seahorse data were analyzed with the Wave software (Agilent Technologies), including the normalization to cell numbers, and the data are presented as mpH/min/cell for ECAR and as pmol/min/cell for OCR or as a ratio to the noninfected or nontreated cells.

### Measurement of lactate production

Cells (same number as ATP assay) were treated or infected for the indicated time, so that more than 75% of the cells were infected. Plates were then centrifuged for 5 min at 250×*g*, then 56 μl of medium or lactate standard (Sigma-Aldrich) was used for the assay. Lactate produced was measured using a colorimetric lactate detection assay as described in (55) with the following reagents: nicotinamide adenine dinucleotide (14.3 mM NAD+, Sigma-Aldrich), 0.52 M hydrazine (Sigma-Aldrich) both prepared in assay buffer (0.50 M Glycine (Sigma-Aldrich) and 2.5 mM, EDTA, pH 9.5), LDH (19 U/ml, Sigma-Aldrich), 9.0 M urea buffer. Absorbance at 340 nm was measure with Cytation 5 Cell Imaging Multi-Mode Reader (BioTek). A lactate standard curve (0–4.6 μmole) was used to calculate lactate concentrations. For normalization to protein levels in Figure S4, cells were lysed in 2 M urea buffer and proteins level was measured with Pierce BCA protein assay kit (Thermo Fisher Scientific), as indicated by the manufacturer. For normalization to cell number in Figure 3*E* and S3*D*. cells were counted by microcopy as for the metabolic flux analyses.

### RNA interference

For siRNA experiments, 5 × 10^4^ HeLa cells were mixed with Lipofectamine RNAiMAX (Invitrogen), following the manufacturer’s recommendation, with 10 nM siRNA, before being plated in a 24-well plate. The culture medium was changed the following day and progeny assay were performed 2 days post treatment with the siRNA. Downregulation efficiency was verified by performing a lactate assay. Two sets of siRNA against GPI were used and their sequences are as follows: siGPI1-sense: 5′-CCAAGCUCACACCAUUCAU-3′; siGPI1-antisense: 5′-AUGAAUGGUGUGAGCUUGG-3′; siGPI2-sense: 5′-CAUGGAGUCCAAUGGGAAA-3′; siGPI2-antisense: 5′-UUUCCCAU UGGACUCCAUG-3′. Control siRNAs (#SR-CL000-005) were purchased from Eurogentec (Belgium).

### Statistical analyses

The R environment v4.0.5 was used for all the analyses except Figure 2*E*, which was analyzed by performing an Ordinary 2-way ANOVA with a Tukey's multiple comparison test (56). Data were neither averaged nor normalized prior analyses. Data were fitted to a linear model that included the variable of interest and the experiment date as covariate, but only when technical replicates were available (*i.e.*, more than 1 value per variable of interest and per experiment date). Two by 2 effect comparisons (contrast comparisons) were performed with the emmeans() function of the emmeans package. Unequal variance *t* test (Welch test) was used in bivariate designs. Statistical significance was set to *p* ≤ 0.05. In each figure, type I error was controlled by correcting the *p* values according to the Benjamini & Hochberg method (“BH” option in the p.adjust() function of R). Results are detailed in Table S1.

## Data availability

All data are contained in the article.

## Supporting information

This article contains supporting information.

## Conflict of interest

The authors declare that they have no conflict of interest with the contents of this article.
