# Supplementary material for: Chlamydia trachomatis development requires both host glycolysis and oxidative phosphorylation but has only minor effects on these pathways
Source: J Biol Chem. 2022 Aug 2;298(9):102338. doi: 10.1016/j.jbc.2022.102338 (PMC9449673; doi:10.1016/j.jbc.2022.102338)
Supplement: Supplementary file 1 — Supplemental Table S1 [file mmc1.pdf]

Table S1. Statistical analyses

The R environment v4.0.5 was used for all the analyses except Fig. 2.E for which an Ordinary 2-way ANOVA with a Tukey's multiple comparison test was performed.

Figure	Comparison	Alternative hypothesis	Sample size	Test	p-value	Adjusted p-value
1.B	CTD and CTDm	mean difference	n = 76, n = 76	Welch test	0,02	*nr*
1.C	CTD and CTDm	mean difference	n = 3, n = 3	Welch test	0,03	*nr*
1.D	CTD and CTDm	effect difference	n = 9, n =9 | df = 13	Contrast test in linear model	2e-5	*nr*
1.E	CTD and CTDm	effect difference	n = 9, n =9 | df = 14	Contrast test in linear model	1e-6	*nr*
2.A	Infected 24 h and Not infected	effect difference	n = 14, n =14 | df = 78	Contrast test in linear model	0.29	0.57
	Infected 30 h and Not infected	effect difference	n = 14, n =14 | df = 78	Contrast test in linear model	0.65	0.65
	Infected 48 h and Not infected	effect difference	n = 14, n =14 | df = 78	Contrast test in linear model	0.39	0.58
2.C	Infected 8 h and Not infected	effect difference	n = 12, n =12 | df = 42	Contrast test in linear model	0.51	0,51
	Infected 30 h and Not infected	effect difference	n = 12, n =12 | df = 42	Contrast test in linear model	1e-5	2e-5
2.D	NaN3/2-DG and Not treated	effect difference	n = 8, n =8 | df = 11	Contrast test in linear model	1e-5	*nr*
3.A	Not treated and 24h	mean difference	n = 3, n = 3	Welch test	0.02	0.06
	Not treated and 30h	mean difference	n = 3, n = 3	Welch test	0.08	0.08
	Not treated and 48h	mean difference	n = 3, n = 3	Welch test	0.04	0.06
3.C	Infected 24 h and Not infected	effect difference	n = 11, n =13 | df = 74	Contrast test in linear model	0.60	0.79
	Infected 30 h and Not infected	effect difference	n = 16, n =13 | df = 74	Contrast test in linear model	0.12	0.35
	Infected 48 h and Not infected	effect difference	n = 13, n =13 | df = 74	Contrast test in linear model	0.79	0.79
3.D	Infected 24 h and Not infected 24 h	effect difference	n = 20, n =20 | df = 110	Contrast test in linear model	0.22	0.32
	Infected 30 h and Not infected 30 h	effect difference	n = 20, n =20 | df = 110	Contrast test in linear model	0.53	0.53
	Infected 48 h and Not infected 48 h	effect difference	n = 20, n =20 | df = 110	Contrast test in linear model	0.03	0.09
3.E	Infected 24 h and Not infected 24 h	effect difference	n = 20, n =20 | df = 110	Contrast test in linear model	1e-4	2e-4
	Infected 30 h and Not infected 30 h	effect difference	n = 20, n =20 | df = 110	Contrast test in linear model	0.09	0.09
	Infected 48 h and Not infected 48 h	effect difference	n = 20, n =20 | df = 110	Contrast test in linear model	8e-5	2e-4
2.E	Infected 24 h and Not infected	effect difference	n = 10, n =10 | df = 53	Contrast test in linear model	0.30	0.45
	Infected 30 h and Not infected	effect difference	n = 11, n =10 | df = 53	Contrast test in linear model	0.06	0.18
	Infected 48 h and Not infected	effect difference	n = 11, n =10 | df = 53	Contrast test in linear model	0.62	0.62
3.F	Infected 24 h and Not infected	mean difference	n = 3, n = 3	Welch test	0.77	0.77
	Infected 30 h and Not infected	mean difference	n = 3, n = 3	Welch test	0.76	0.77
	Infected 48 h and Not infected	mean difference	n = 3, n = 3	Welch test	0.65	0.77
3.G	Not treated and 24h	mean difference	n = 3, n = 3	Welch test	0.90	0.91
	Not treated and 30h	mean difference	n = 3, n = 3	Welch test	0.80	0.91
	Not treated and 48h	mean difference	n = 3, n = 3	Welch test	0.85	0.91
3.H	Not treated and 24h	mean difference	n = 4, n = 4	Welch test	0.35	0.60
	Not treated and 30h	mean difference	n = 4, n = 4	Welch test	0.40	0.60
	Not treated and 48h	mean difference	n = 4, n = 4	Welch test	0.98	0.98
3.I	Not treated and 24h	mean difference	n = 4, n = 4	Welch test	0.94	0.98
	Not treated and 30h	mean difference	n = 4, n = 4	Welch test	0.98	0.98
	Not treated and 48h	mean difference	n = 4, n = 4	Welch test	0.93	0.98
4.A	0 and 5	mean difference	n = 3, n = 3	Welch test	0.24	0.26
	0 and 10	mean difference	n = 3, n = 3	Welch test	4e-3	0.01
	0 and 25	mean difference	n = 3, n = 3	Welch test	2e-7	1e-6
4.B	0 and 0.5	mean difference	n = 3, n = 3	Welch test	3e-3	3e-3
	0 and 1	mean difference	n = 3, n = 3	Welch test	1e-3	2e-3
	0 and 2	mean difference	n = 3, n = 3	Welch test	5e-4	2e-3
4.C	0 and 10	mean difference	n = 3, n = 3	Welch test	1e-3	1e-3
	0 and 20	mean difference	n = 3, n = 3	Welch test	9e-4	1e-3
	0 and 40	mean difference	n = 3, n = 3	Welch test	4e-3	1e-3
4.D	0 and 0.5	effect difference	n = 12, n =12 | df = 52	Contrast test in linear model	1e-13	3e-13
	0 and 2	effect difference	n = 12, n =12 | df = 52	Contrast test in linear model	6e-12	8e-12
	0 and 10	effect difference	n = 12, n =12 | df = 52	Contrast test in linear model	4e-8	4e-8
	0 and 40	effect difference	n = 12, n =12 | df = 52	Contrast test in linear model	1e-14	5e-14
4.E	0 and 5	mean difference	n = 3, n = 3	Welch test	0.62	0.62
	0 and 10	mean difference	n = 3, n = 3	Welch test	0.46	0.62
	0 and 25	mean difference	n = 3, n = 3	Welch test	0.10	0.32
4.F	0 and 5	mean difference	n = 3, n = 3	Welch test	0.23	0.23
	0 and 10	mean difference	n = 3, n = 3	Welch test	0.02	0.03
	0 and 25	mean difference	n = 3, n = 3	Welch test	0.01	0.03
4.G	Not treated and 0.5	mean difference	n = 4, n = 4	Welch test	0.70	0.91
	Not treated and 1	mean difference	n = 4, n = 4	Welch test	0.86	0.91
	Not treated and 2	mean difference	n = 4, n = 4	Welch test	0.10	0.59
	Not treated and 10	mean difference	n = 4, n = 4	Welch test	0.67	0.91
	Not treated and 20	mean difference	n = 4, n = 4	Welch test	0.78	0.91
	Not treated and 40	mean difference	n = 4, n = 4	Welch test	0.91	0.91
4.J	0 and 5	mean difference	n = 3, n = 3	Welch test	2e-3	2e-3
	0 and 10	mean difference	n = 3, n = 3	Welch test	8e-5	2e-4
4.K	0 and 0.5	mean difference	n = 3, n = 3	Welch test	3e-3	3e-3
	0 and 1	mean difference	n = 3, n = 3	Welch test	3e-4	6e-4
5.A	0 and 5	mean difference	n = 4, n = 4	Welch test	0.69	0.92
	0 and 10	mean difference	n = 4, n = 4	Welch test	0.54	0.92
	0 and 25	mean difference	n = 4, n = 4	Welch test	0.92	0.92
5.C	Not.treated - siGPI1	mean difference	n = 4, n = 4	Welch test	0.49	0.77
	Not.treated - siGPI2	mean difference	n = 4, n = 4	Welch test	0.52	0.77
	siGPI1 - siGPI2	mean difference	n = 4, n = 4	Welch test	0.93	0.93

5.E	GPI.KO26 - GPI.KO8	mean difference	n = 3, n = 3	Welch test	0.93	0.93
	GPI.KO26 - WT	mean difference	n = 3, n = 3	Welch test	0.67	0.93
	GPI.KO8 - WT	mean difference	n = 3, n = 3	Welch test	0.76	0.93
6.A	0 and 2.5	effect difference	n = 12, n =12 | df = 49	Contrast test in linear model	1e-13	3e-13
	0 and 5	effect difference	n = 12, n =11 | df = 49	Contrast test in linear model	6e-12	8e-12
	0 and 10	effect difference	n = 12, n =11 | df = 49	Contrast test in linear model	4e-8	4e-8
	0 and 25	effect difference	n = 12, n =10 | df = 49	Contrast test in linear model	1e-14	5e-14
6.B	0 and 5	effect difference	n = 12, n =11 | df = 41	Contrast test in linear model	1e-9	1e-9
	0 and 10	effect difference	n = 12, n =12 | df = 41	Contrast test in linear model	2e-14	3e-14
	0 and 25	effect difference	n = 12, n =12 | df = 41	Contrast test in linear model	4e-16	1e-15
6.C	0 and 5	effect difference	n = 11, n =10 | df = 36	Contrast test in linear model	8e-3	8e-3
	0 and 10	effect difference	n = 11, n =10 | df = 36	Contrast test in linear model	9e-6	1e-5
	0 and 25	effect difference	n = 11, n =11 | df = 36	Contrast test in linear model	6e-8	2e-7
6.D	0 and 2.5	effect difference	n = 11, n =10 | df = 36	Contrast test in linear model	0.02	0.06
	0 and 5	effect difference	n = 11, n =10 | df = 36	Contrast test in linear model	0.48	0.48
	0 and 10	effect difference	n = 11, n =11 | df = 36	Contrast test in linear model	0.31	0.46
6.E	0 and 20	effect difference	n = 10, n =10 | df = 32	Contrast test in linear model	3e-7	3e-7
	0 and 40	effect difference	n = 10, n =9 | df = 32	Contrast test in linear model	1e-9	2e-9
	0 and 80	effect difference	n = 10, n =9 | df = 32	Contrast test in linear model	4e-11	1e-10
6.F	0 and 5	effect difference	n = 5, n =5 | df = 16	Contrast test in linear model	0.55	0.73
	0 and 10	effect difference	n = 5, n =5 | df = 16	Contrast test in linear model	0.83	0.83
	0 and 20	effect difference	n = 5, n =5 | df = 16	Contrast test in linear model	0.24	0.48
	0 and 40	effect difference	n = 5, n =5 | df = 16	Contrast test in linear model	0.11	0.14
5.G	0 and 0.5	effect difference	n = 11, n =11 | df = 39	Contrast test in linear model	2e-17	3e-17
	0 and 1	effect difference	n = 11, n =11 | df = 39	Contrast test in linear model	6e-17	7e-17
	0 and 2	effect difference	n = 11, n =12 | df = 39	Contrast test in linear model	1e-19	3e-19
6	0 and 0.5	effect difference	n = 12, n =9 | df = 35	Contrast test in linear model	2e-3	2e-3
	0 and 1	effect difference	n = 12, n =10 | df = 35	Contrast test in linear model	1e-7	2e-7
	0 and 2	effect difference	n = 12, n =10 | df = 35	Contrast test in linear model	4e-11	1e-10
6.I	No Glucose and Glucose	effect difference	n = 11, n =11 | df = 18	Contrast test in linear model	2e-11	*nr*
S3.C	Infected 24 h and Not infected 24 h	effect difference	n = 16, n =16 | df = 86	Contrast test in linear model	0.09	0.13
	Infected 30 h and Not infected 30 h	effect difference	n = 15, n =16 | df = 86	Contrast test in linear model	0.75	0.75
	Infected 48 h and Not infected 48 h	effect difference	n = 16, n =16 | df = 86	Contrast test in linear model	8e-4	2e-3
S3.D	Infected 24 h and Not infected 24 h	effect difference	n = 16, n =16 | df = 86	Contrast test in linear model	0.98	0.98
	Infected 30 h and Not infected 30 h	effect difference	n = 15, n =16 | df = 86	Contrast test in linear model	0.15	0.23
	Infected 48 h and Not infected 48 h	effect difference	n = 16, n =16 | df = 86	Contrast test in linear model	1e-6	3e-6
S4.A	0 and 5	effect difference	n = 12, n =12 | df = 53	Contrast test in linear model	4e-4	5e-4
	0 and 10	effect difference	n = 12, n =12 | df = 53	Contrast test in linear model	3e-3	3e-3
	0 and 25	effect difference	n = 12, n =12 | df = 53	Contrast test in linear model	4e-9	9e-9
	0 and 50	effect difference	n = 12, n =12 | df = 53	Contrast test in linear model	4e-9	9e-9
S4.B	0 and 1	mean difference	n = 6, n = 3	Welch test	0.51	0.51
	0 and 2.5	mean difference	n = 6, n = 3	Welch test	0.09	0.12
	0 and 5	mean difference	n = 6, n = 3	Welch test	0.02	0.03
	0 and 10	mean difference	n = 6, n = 3	Welch test	0.01	0.04
S4.C	0 and 10	mean difference	n = 3, n = 3	Welch test	0.45	*nr*
S5.B	0 and 10	effect difference	n = 11, n =12 | df = 40	Contrast test in linear model	6e-4	2e-3
	0 and 20	effect difference	n = 11, n =12 | df = 40	Contrast test in linear model	0.47	0.71
	0 and 40	effect difference	n = 11, n =11 | df = 40	Contrast test in linear model	0.95	0.95
S5.C	0 and 2.5	effect difference	n = 14, n =14 | df = 50	Contrast test in linear model	0.09	0.09
	0 and 5	effect difference	n = 14, n =15 | df = 50	Contrast test in linear model	2e-3	3e-3
	0 and 10	effect difference	n = 14, n =14 | df = 50	Contrast test in linear model	1e-6	4e-6
S5.D	0 and 0.5	effect difference	n = 11, n =9 | df = 35	Contrast test in linear model	9e-6	1e-5
	0 and 1	effect difference	n = 11, n =11 | df = 35	Contrast test in linear model	2e-4	2e-4
	0 and 2	effect difference	n = 11, n =10 | df = 35	Contrast test in linear model	1e-10	3e-10
