# Supplementary material for: Chlamydia trachomatis development requires both host glycolysis and oxidative phosphorylation but has only minor effects on these pathways
Source: J Biol Chem. 2022 Aug 2;298(9):102338. doi: 10.1016/j.jbc.2022.102338 (PMC9449673; doi:10.1016/j.jbc.2022.102338)
Supplement: Supplementary file 2 — Supplemental Figure S1–S5 [file mmc2.pdf]

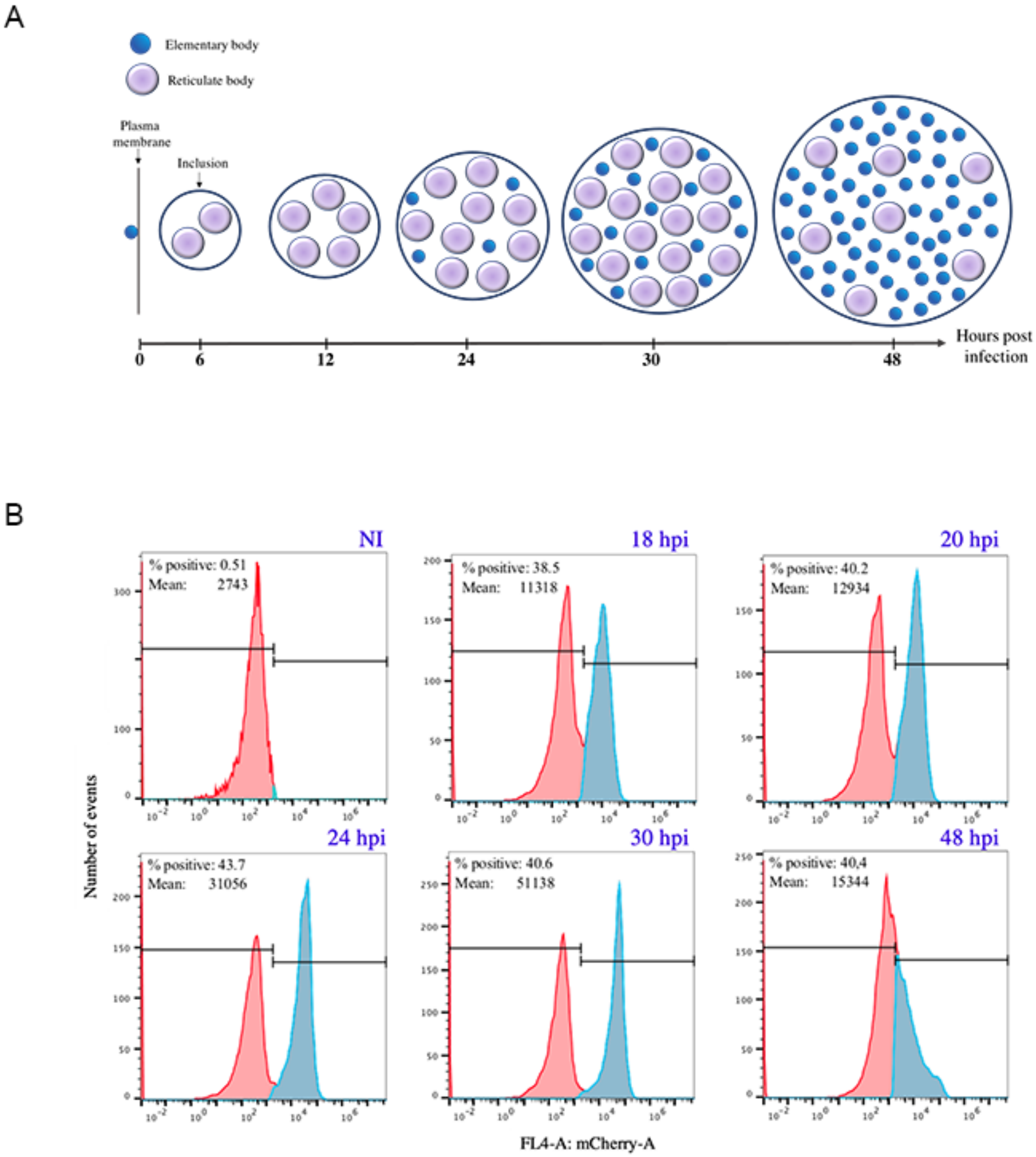

Figure S1. *C. trachomatis* developmental cycle and bacterial load monitoring by flow cytometry (A) Schematic view of the developmental cycle of *C. trachomatis*. (**B**) Quantification of mCherry expressing *C. trachomatis* D/UW-3/CX (CTDm) over time. Hela cells were infected with CTDm and fixed 18, 20, 24, 30 and 48 hpi. Samples were analyzed through flow cytometry in the red channel (FL4-A). mCherry negative cells are represented in red and the positive cells are in blue. The percentage of infection (out of total cells) and the mCherry mean fluorescence of the positive population (average signal per cell) are displayed on the graphs. This experiment is representative of two. Note that at 48 hpi reinfection events have occurred, precluding the distinction between the infected and non-infected population. NI = non-infected.

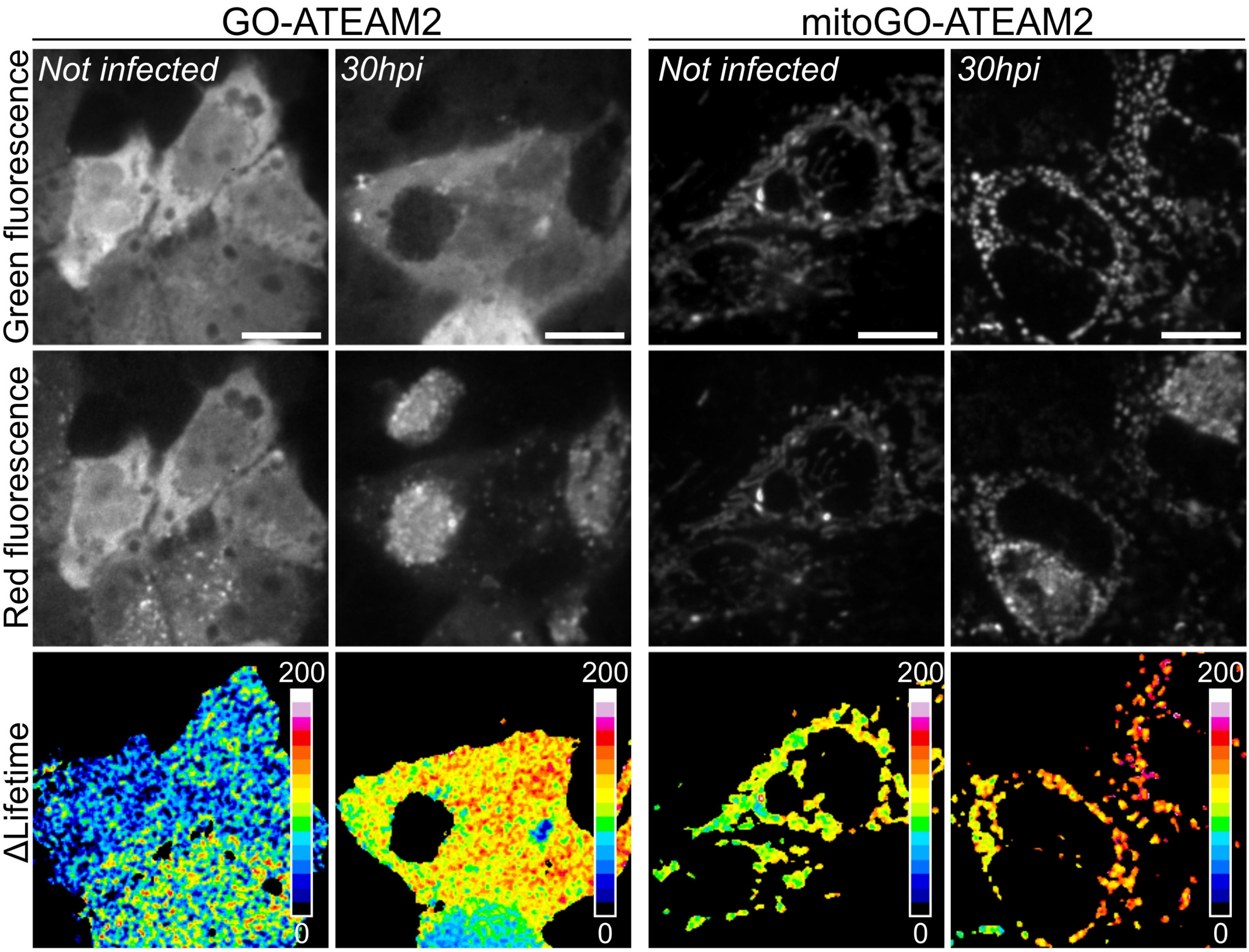

Figure S2: Measure of subcellular ATP levels using fluorescent probes. HeLa cells were transfected with plasmids expressing GO-ATEAM2 (left) and mitoGO-ATEAM2 (right) for 24 h and infected with CTDm or not before fixation 30 hpi. Cells were excited as described in the method section and imaged in both green and red channels, corresponding to the FRET donor and acceptor, respectively. The shift in ΔLifetime is represented in the bottom panel as a heat map. Bar is 10 μm.

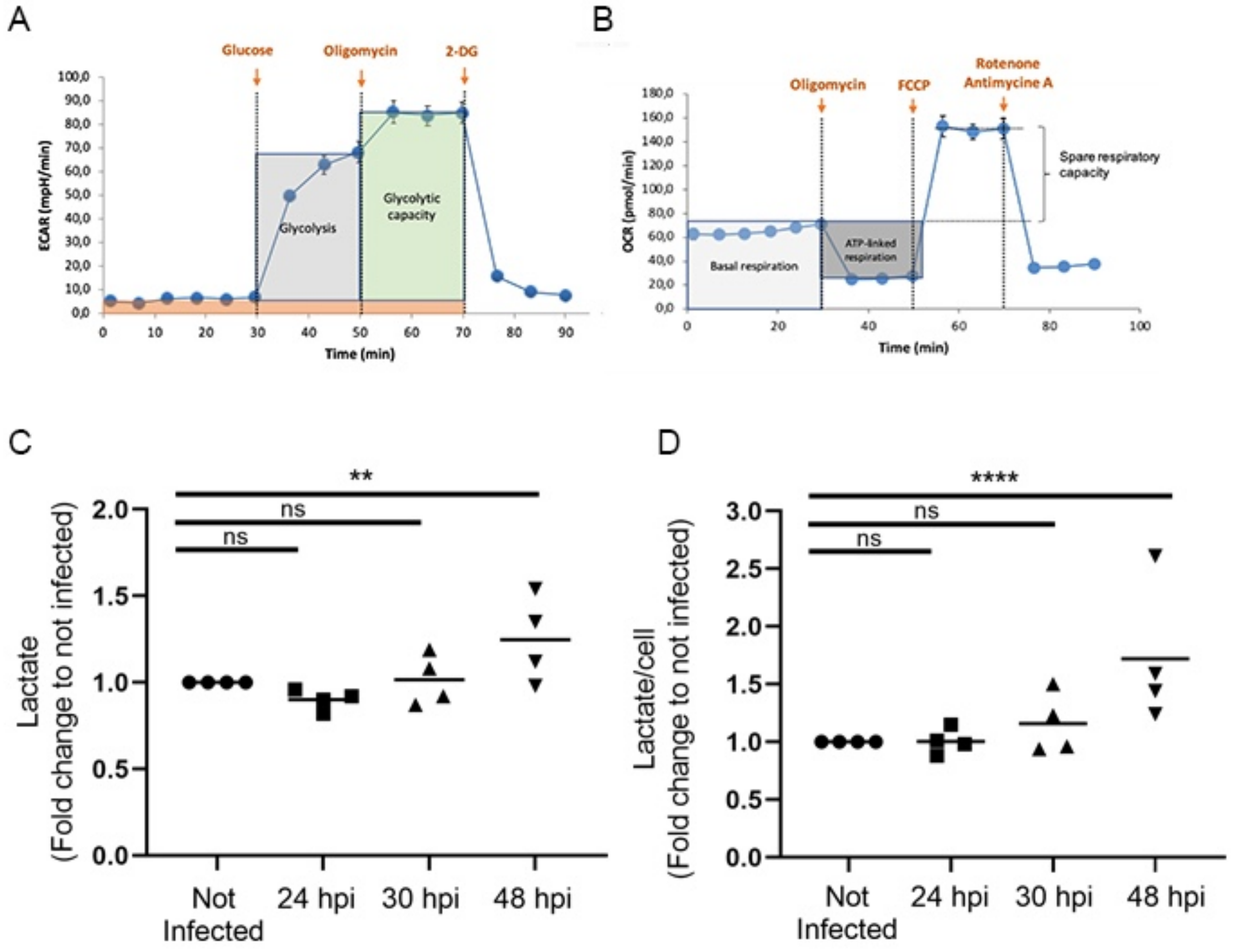

Figure S3 Glycolysis and Mito stress test profiles (**A**) Glycolysis stress test profile of one representative experiment using untreated A2EN cells. Sequential injections of the indicated compounds measure glycolysis (grey box), glycolytic capacity (green box), non-glycolytic acidification (orange box). ECAR values displayed in the main figures correspond to the "Glycolysis" measurements. In A2EN cells, the glycolysis values were close to the total glycolytic capacity measured upon oligomycin addition. (**B**) Mito stress test profile of one representative experiment using untreated A2EN cells. OCR values displayed in the main figures correspond to the "ATP-linked respiration" measurements. (**C, D**) HeLa cells were infected with CTDm for 24, 30 and 48 hpi after what the concentration of lactate in the culture medium was measured, without (**C**) or with (**D**) normalization to the cell number.

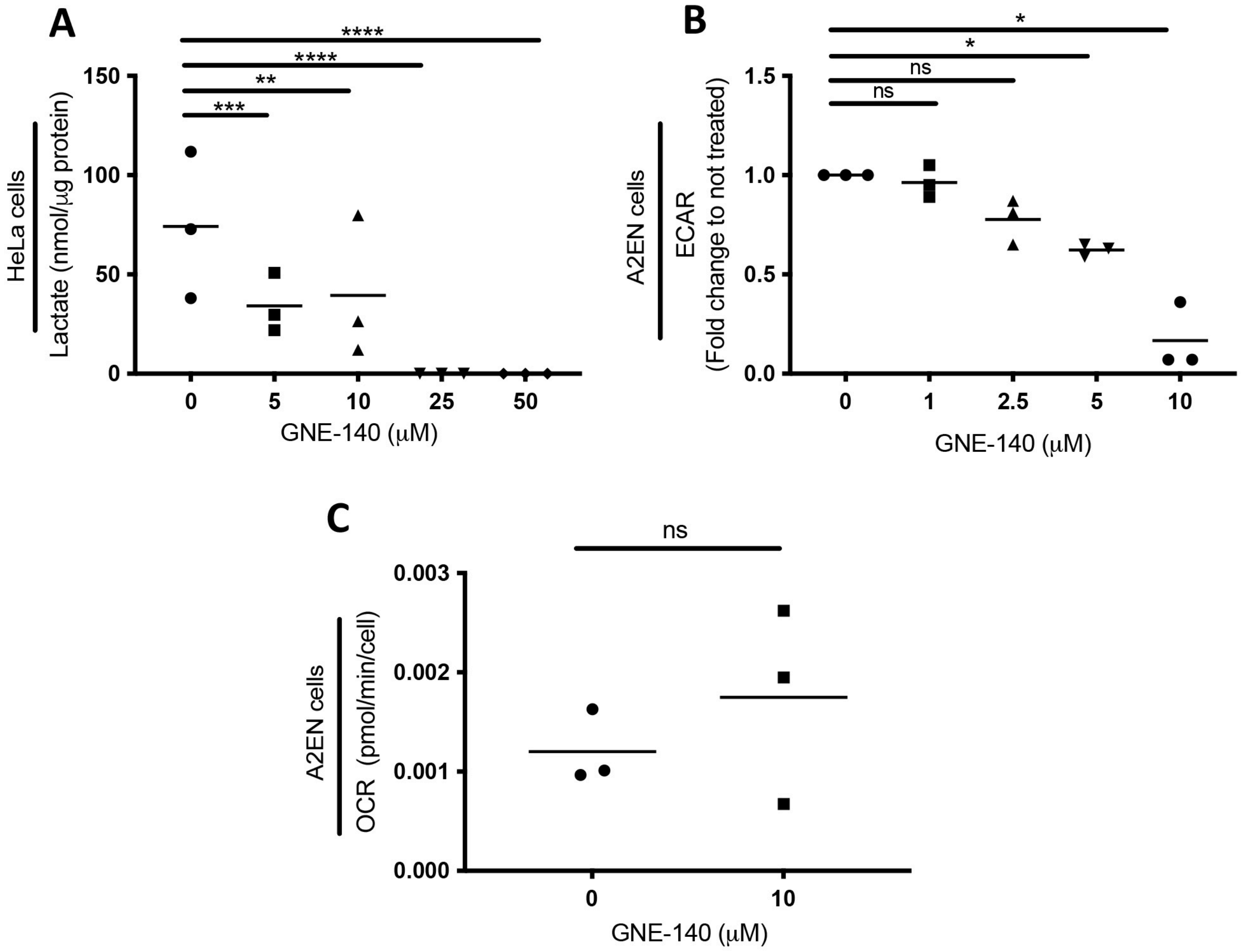

Figure S4. GNE-140 inhibits lactate production without affecting OxPhos (**A**) Hela cells were treated with indicated GNE-140 concentrations and 24 h later lactate production was measured in the medium as described in the methods. (**B**) A2EN cells were treated with indicated GNE-140 concentrations for 24 h then ECAR was measured as described in the methods. (**C**) A2EN cells were treated or not for 24 h with GNE-140 before performing a Mito stress test. For each panel, the results of three independent experiments and the mean are displayed.

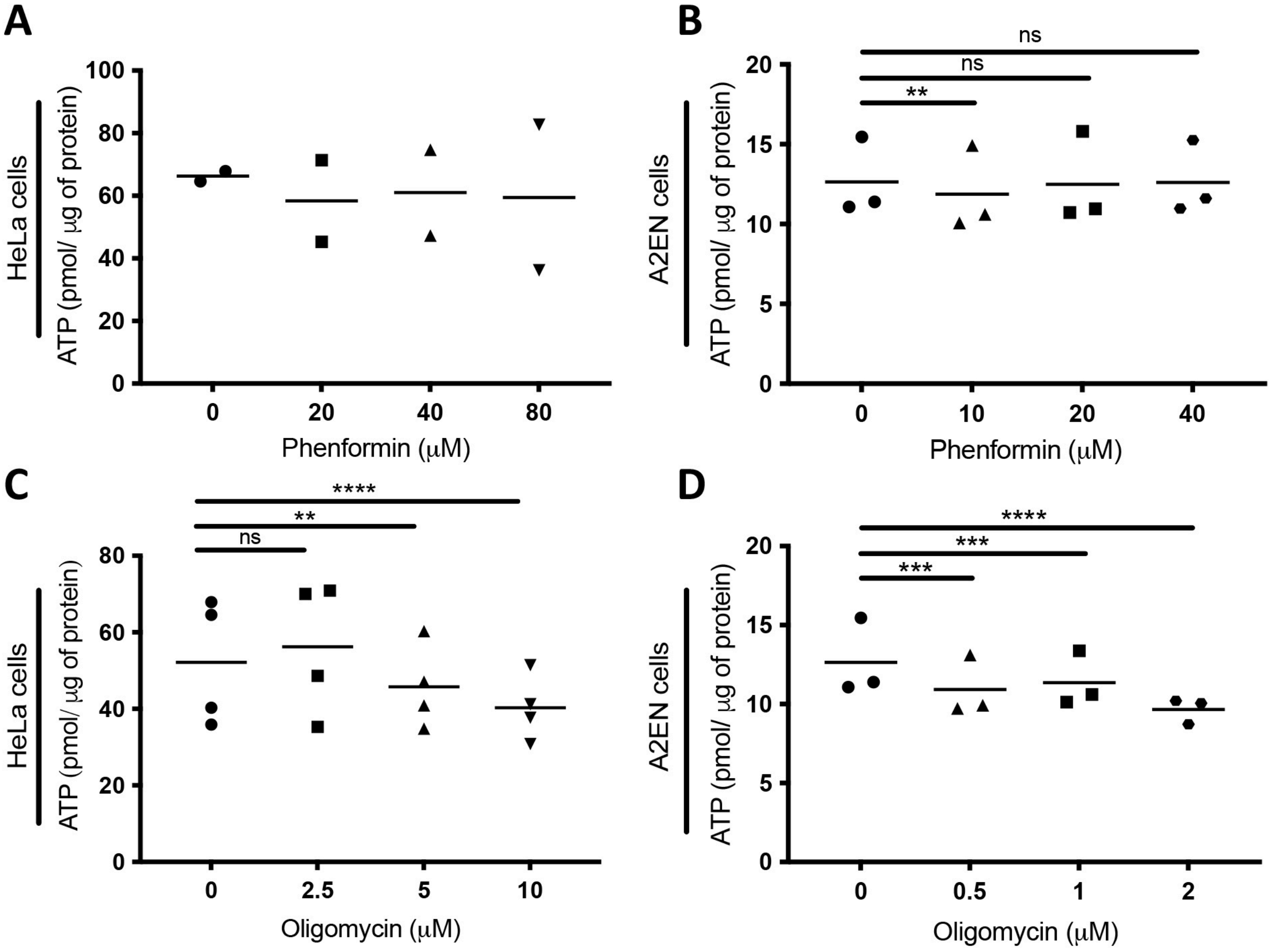

Figure S5. ATP levels are differentially affected by GNE-140 and OxPhos inhibitors in primary and HeLa cells. HeLa cells (**A**,**C**) and A2EN cells (**B**,**D**) were treated with the indicated concentrations of phenformin or oligomycin for 2 h before measuring ATP levels as described in the methods. For each panel, the results of three independent experiments and the mean are displayed.
